# Single‐cell RNA‐seq reveals novel interaction between muscle satellite cells and fibro‐adipogenic progenitors mediated with FGF7 signalling

**DOI:** 10.1002/jcsm.13484

**Published:** 2024-05-16

**Authors:** Lu Ma, Yingying Meng, Yalong An, Peiyuan Han, Chen Zhang, Yongqi Yue, Chenglong Wen, Xin'e Shi, Jianjun Jin, Gongshe Yang, Xiao Li

**Affiliations:** ^1^ Key Laboratory of Animal Genetics, Breeding and Reproduction of Shaanxi Province, College of Animal Science and Technology Northwest A&F University Xianyang China

**Keywords:** FGF7–FGFR2, muscle regeneration, satellite cells, senescence, single‐cell RNA‐seq

## Abstract

**Background:**

Muscle satellite cells (MuSCs) exert essential roles in skeletal muscle adaptation to growth, injury and ageing, and their functions are extensively modulated by microenvironmental factors. However, the current knowledge about the interaction of MuSCs with niche cells is quite limited.

**Methods:**

A 10× single‐cell RNA sequencing (scRNA‐seq) was performed on porcine *longissimus dorsi* and *soleus* (SOL) muscles to generate a single‐cell transcriptomic dataset of myogenic cells and other cell types. Sophisticated bioinformatic analyses, including unsupervised clustering analysis, marker gene, gene set variation analysis (GSVA), AUCell, pseudotime analysis and RNA velocity analysis, were performed to explore the heterogeneity of myogenic cells. CellChat analysis was used to demonstrate cell–cell communications across myogenic cell subpopulations and niche cells, especially fibro‐adipogenic progenitors (FAPs). Integrated analysis with human and mice datasets was performed to verify the expression of FGF7 across diverse species. The role of FGF7 on MuSC proliferation was evaluated through administering recombinant FGF7 to porcine MuSCs, C2C12, cardiotoxin (CTX)‐injured muscle and d‐galactose (d‐gal)‐induced ageing model.

**Results:**

ScRNA‐seq totally figured out five cell types including myo‐lineage cells and FAPs, and myo‐lineage cells were further classified into six subpopulations, termed as RCN3^+^, S100A4^+^, ID3^+^, cycling (MKI67^+^), MYF6^+^ and MYMK^+^ satellite cells, respectively. There was a higher proportion of cycling and MYF6^+^ cells in the SOL population. CellChat analysis uncovered a particular impact of FAPs on myogenic cells mediated by FGF7, which was relatively highly expressed in SOL samples. Administration of FGF7 (10 ng/mL) significantly increased the proportion of EdU^+^ porcine MuSCs and C2C12 by 4.03 ± 0.81% (*P* < 0.01) and 6.87 ± 2.17% (*P* < 0.05), respectively, and knockdown of FGFR2 dramatically abolished the pro‐proliferating effects (*P* < 0.05). In CTX‐injured muscle, FGF7 significantly increased the ratio of EdU^+^/Pax7^+^ cells by 15.68 ± 5.45% (*P* < 0.05) and elevated the number of eMyHC^+^ regenerating myofibres by 19.7 ± 4.25% (*P* < 0.01). Under d‐gal stimuli, FGF7 significantly reduced γH2AX^+^ cells by 17.19 ± 3.05% (*P* < 0.01) in porcine MuSCs, induced EdU^+^ cells by 4.34 ± 1.54% (*P* < 0.05) in C2C12, and restored myofibre size loss and running exhaustion in vivo (all *P* < 0.05).

**Conclusions:**

Our scRNA‐seq reveals a novel interaction between muscle FAPs and satellite cells mediated by FGF7–FGFR2. Exogenous FGF7 augments the proliferation of satellite cells and thus benefits muscle regeneration and counteracts age‐related myopathy.

## Introduction

Skeletal muscle is a highly heterogeneous tissue that is composed of multinucleated myofibres, muscle satellite cells (MuSCs) and non‐myogenic cells, including fibro‐adipogenic progenitors (FAPs), endothelial cells, immune cells, tenocytes and neurocytes.^1^ MuSCs localized between the muscle fibre and its basal lamina are quiescent in unperturbed adult skeletal muscle and become activated, proliferate and then undergo differentiation and fusion into multinucleated myofibres, in response to adaptive changes, such as growth, acute injury and ageing.^2^ MuSCs are heterogeneous populations that can be distinguished based on unique gene expression and metabolic profiles.^3^

The physiological functions of MuSCs are extensively regulated by niche cells, including myofibres of different types (fast and slow) and other non‐myogenic cells, through intercellular interactions in response to muscle injury, ageing and so forth.^4^, ^5^ Myofibre‐derived granulocyte colony‐stimulating factor (G‐CSF) is required for the maintenance of PAX7^High^ satellite cell (SC) subpopulation in aged mice by regulating asymmetric MuSCs division.^S1^ During acute muscle injury, stromal cell‐derived factor‐1α (SDF1α), derived from FAPs, interacts with CXCR4 on MuSCs and improves early activation and proliferation of MuSCs.^S2^ The loss of WNT1‐inducible signalling pathway protein 1 (WISP1), which is also majorly produced by FAPs, leads to MuSCs dysfunction in aged skeletal muscles.^6^ These findings highlighted the importance of microenvironment on the maintenance and function of MuSCs.

Single‐cell RNA (scRNA) sequencing (scRNA‐seq) provides a powerful tool to unravel a panoramic view of cell–cell communication across various cell types. Recently, scRNA‐seq has been applied to explore heterogeneity in human^S3^ and mice^S4^ skeletal muscle. Porcine muscle (also referred to as pork) provides an important source of dietary protein for humans, and pigs have been widely used as models for various human conditions and diseases, including diseases related to xenotransplantation and muscle atrophy induced by sepsis, due to the similarities in organ size and physiology.^7^, ^8^ Here, muscle tissues of *longissimus dorsi* (LD, majorly composed of fast‐twitch myofibres) and *soleus* (SOL, contained more slow‐twitch myofibre compared with other parts) from 3‐day‐old piglets were subjected to 10× scRNA‐seq. Our work explores the heterogeneity of myogenic cells and reveals a novel interaction between MuSCs and FAPs mediated by FGF7–FGFR2, and exogenous FGF7 facilitates proliferation of myogenic SCs and thus benefits muscle regeneration and counteracts age‐related myopathy.

## Methods

### Ethics declarations

Animal handling and treatment were approved by the Committee of Experimental Animal Management at Northwest A&F University (approval ID: NWAFU‐314035248).

### Cell isolation

Muscle samples of 3‐day‐old male piglets were digested with 1‐mg/mL collagenase type II (Invitrogen) for 40 min and subsequently 0.25% trypsin (Thermo Fisher Scientific) for 30 min at 37°C. Dissociated cells were then filtered through a 200‐mesh strainer and a 70‐μm strainer sequentially.

### Single‐cell RNA sequencing and data analysis

Single cells were isolated from LD and SOL muscles in 3‐day‐old Duroc × Landrace × Yorkshire littermate piglets (a pool of three pigs) and loaded in the 10x Genomics Chromium v3.0 platform (Pleasanton, CA, USA). Library sequencing was performed on an Illumina HiSeq 2500 (BGI Shenzhen, Guangdong, China), according to the manufacturer's guidelines (10x Genomics). Raw data were processed by Cell Ranger 6.0.0 (10x Genomics). Alignment (to the Sscrofa11.1 genome), filtering, barcode and unique molecular identifier (UMI) counting were performed with the Cell Ranger count module. Processed data were analysed using Seurat 4.3.0 and normalized with the ‘LogNormalize’ method.^S5^ Doublets were evaluated and removed with scDblFinder 1.15.3.^S6^ ^,^ ^S7^ Cells with mitochondrial abundance >8% were removed. Integration of data from different samples was performed using Harmony 0.1.1.^S8^ Pseudo‐temporal analysis was conducted with Monocle 3.^S9^ RNA velocity was analysed with scVelo 0.2.5 in Python 3.9.1.^S10^ Pathway activity was assessed in individual cells using GSVA 1.40.1 with the ‘gsva’ method.^S11^ AUCell (1.14.0) was performed to assess enrichment scores for specific gene sets in scRNA data.^S12^ CellChat 1.6.1 was employed to infer all cell–cell communications across all cell types.^S13^

### Data availability

Porcine muscle scRNA‐seq dataset has been deposited at PRJNA1077076. Several public datasets were used, including mice muscle stem cell RNA‐seq (GSE103164), human myogenic cell scRNA‐seq (GSE188215), human muscle scRNA‐seq (GSE130646) and mice muscle scRNA‐seq (GSE143435) datasets.

### Cell culture

Differential adherence was performed to increase the purity of porcine MuSCs. Briefly, single‐cell suspensions from muscle digests were seeded on plates, and the supernatant was collected as P0. Differential adherence was repeated with a half‐hour interval until P4, which was used in the following experiments.

Porcine MuSCs were cultured with the growth medium containing 20% foetal bovine serum (FBS), basic fibroblast growth factor (bFGF) (5 ng/mL, Novoprotein, GMP‐C046), 1% GlutaMAX (Thermo Fisher Scientific, 35050‐061), 1% non‐essential amino acid (NEAA) (Thermo Fisher Scientific, 1140‐050), chicken embryo extract (CEE) (1.5 mg/500 mL, GEMINI, 100‐1634), and 1% penicillin and streptomycin with RPMI Medium 1640 basic.

C2C12 cells were cultured in growth medium comprised of high‐glucose Dulbecco's modified Eagle's medium (DMEM) (H30022.01, HyClone, CT, USA) supplemented with 10% FBS and 1% penicillin/streptomycin.

### Transfection of small interfering RNA

Small interfering RNAs (siRNAs) were designed and synthesized by RiboBio (Guangzhou, China) and transfected with Lipofectamine 2000 (Lot 2462811, Thermo Fisher Scientific) for 8–12 h following the manufacturer's conditions. The si‐pFGFR2 (porcine) sequences were as follows: 5′‐CCACUGGUGAGGAUUACAATT‐3′ (sense) and: 5′‐UUGUAAUCCUCACCAGUGGTT‐3′ (antisense). The si‐mFGFR2 (mice) sequences were as follows: 5′‐AGCCCUGUUUGAUAGAGUAUATT‐3′ (sense) and 5′‐UAUACUCUAUCAAACAGGGCUTT‐3′ (antisense).

### 5‐Ethynyl‐2′‐deoxyuridine treatment and detection

Cell proliferation assay was determined with the Cell‐Light 5‐Ethynyl‐2′‐Deoxyuridine (EdU) Apollo 567 In Vitro Kit (C10310‐1, RiboBio, Guangzhou, China). Porcine MuSCs and C2C12 were incubated with EdU‐A reagent for 4 and 2 h at 37°C, respectively, and signals were detected according to the manufacturer's instructions. EdU^+^ cells in skeletal muscle sections were detected with the Cell‐Light Apollo 488 Stain Kit (C10371‐3, RiboBio).

### d‐Galactose‐induced cell senescence

According to previous studies,^S14^ ^,^ ^S15^ different doses of d‐galactose (d‐gal: 0, 1, 5, 10, 20 and 40 g/L; CAS: 59‐23‐4, high‐performance liquid chromatography [HPLC] ≥98%, HY‐N0210, MedChemExpress) were assessed in porcine MuSCs and C2C12, and 20‐g/L d‐gal for 48 h was used to induce cell senescence.

### Muscle injury and regeneration in mice

Male C57BL/6 mice at 8 weeks old were purchased from the Laboratory Animal Center of Xi'an Jiaotong University, randomly allocated to the indicated groups and housed with a 12‐h dark/light cycle with ad libitum access to water and food.

An agent of 50‐μL cardiotoxin (CTX, 10 μM in 0.9% NaCl; HF005, Hengfei Biotechnology, Shanghai, China) was injected in *tibialis anterior* (TA) muscles to induce injury according to previous studies.^S16^ ^,^ ^S17^ Mouse recombinant FGF7 protein (0.5 μg in 50 μL of 0.9% NaCl; 5028‐KG‐025, R&D Systems, Bio‐Techne, Minneapolis, MN, USA) was injected into TA muscle at 1 day after injury. Prior to each intramuscular injection, mice were anaesthetized with 1.25% tribromoethanol (0.15 mL/10 g body weight; M2910, Nanjing Aibei Biotechnology Co., Ltd). Mice were subjected to intraperitoneal injections of EdU (50 mg/kg body weight; HY‐118411, MedChemExpress) for two consecutive days before being analysed. TA samples were collected at 0, 3, 5 and 15 days after injury.

### d‐Galactose‐induced mouse ageing

An ageing model was generated with continuous intraperitoneal injection of d‐gal (150 mg/kg/day) for 6 weeks, according to the literature,^9^ and recombinant FGF7 was injected into TA muscle four times within the last 2 weeks. A treadmill exhaustion test was performed after three injections of FGF7, according to previous reports.^S18^ ^,^ ^S19^ Samples were collected 1 day after the fourth injection of FGF7. CTX‐induced TA injury was performed the following day after the end of the d‐gal injection.

### Sample preparation for histological analysis

For haematoxylin and eosin (H&E) and embryonic myosin heavy chain (eMyHC) staining of CTX‐injured muscles, frozen sections and a half‐hour fixation with 4% paraformaldehyde (Servicebio) procedure were applied. For other histological analysis, fresh muscles were fixed with 4% paraformaldehyde for over 24 h and subjected to paraffin sectioning. The myofibre cross‐sectional area (CSA) was calculated using ImageJ for ~5 fields of each view and 5 views for each.

### Immunofluorescence

Muscle sections and cells were fixed with 4% paraformaldehyde for over 30 min, permeabilized with 0.2% Triton X‐100 for 30 min, blocked with 5% bovine serum albumin in phosphate‐buffered saline (PBS) for 2 h, incubated with primary antibodies at 4°C overnight and secondary antibodies for 2 h at room temperature, washed with PBS and incubated with DAPI (C0060, Solarbio, Beijing, China) for 10 min. Information about the antibodies is listed in *Table* *S1*.

### Western blot

Proteins were extracted from muscle samples and cultured cells using radioimmunoprecipitation assay (RIPA) lysis buffer (Beyotime, Shanghai, China) supplemented with protease and phosphatase inhibitors (1:100, CWBio, Beijing, China) and were quantified with a bicinchoninic acid (BCA) protein assay kit (Thermo Fisher Scientific). Western blotting analyses were conducted according to the reported method.^S20^ The antibodies used were listed in *Table* *S1*.

### Statistical analysis

All data were presented as mean ± standard deviation (SD). Statistical analysis was performed by GraphPad Prism 9.5.0. Differences between two groups were assessed using an unpaired Student's *t* test. Differences among three or more groups were evaluated using a one‐way analysis of variance (ANOVA). *P* < 0.05 was set as statistical significance.

## Results

### Profiling of skeletal muscle mononuclear cells in pigs

**Figure 1:**
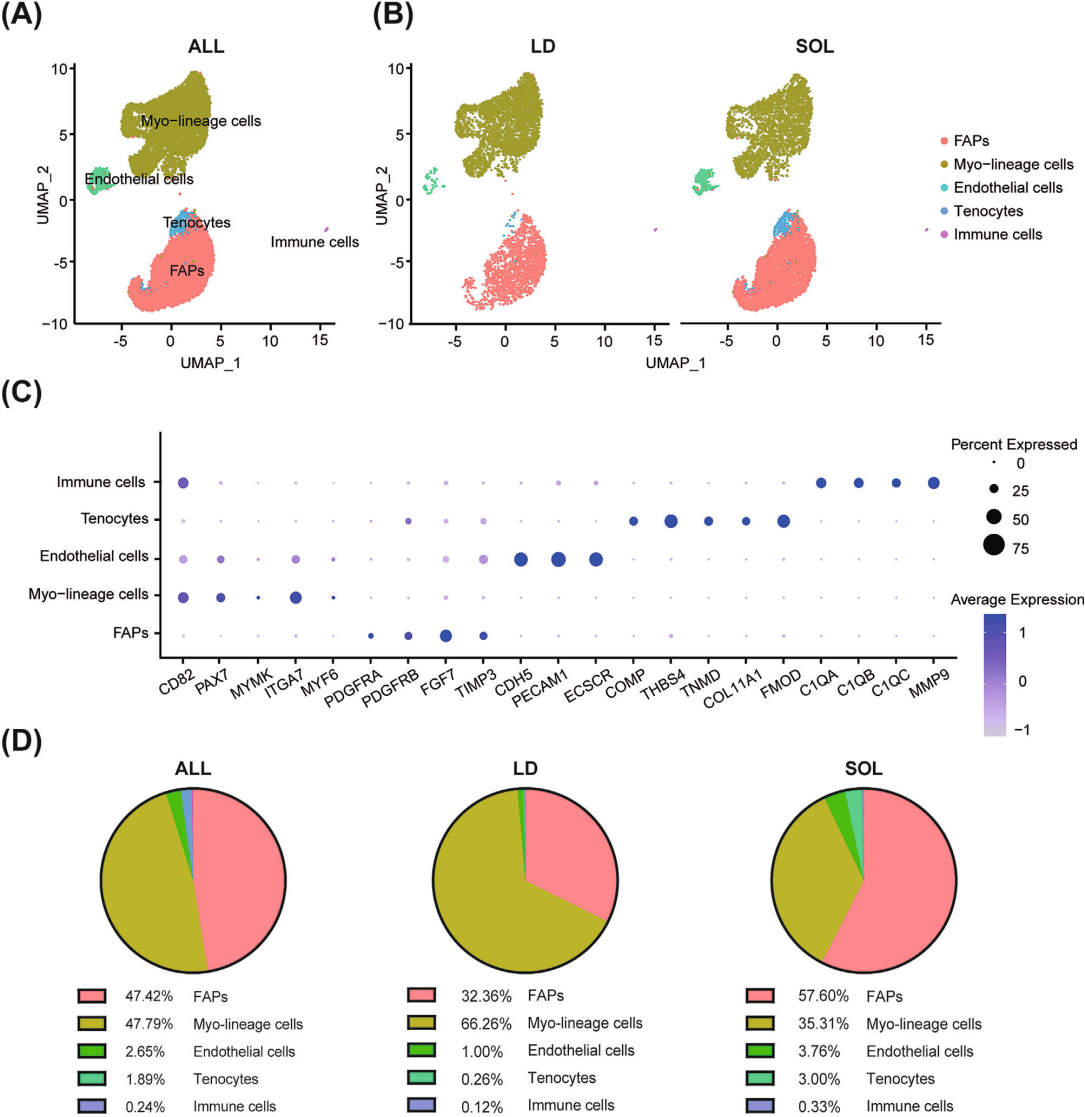
Profiling of porcine skeletal muscle mononuclear cells. (A) Mononuclear cells of porcine *longissimus dorsi* (LD) and *soleus* (SOL) muscles were isolated and subjected to 10× scRNA‐seq analysis. UMAP plot labelled by five distinct cell types of porcine skeletal muscle mononuclear cells. (B) UMAP plots of porcine muscle mononuclear cells split by LD and SOL muscles. (C) Dot plot showing differently expressed marker genes associated with five cell types. (D) Pie graphs of the proportion of five cell types in all samples and individual muscles (LD and SOL).

Mononuclear cells from the digests of LD and SOL from 3‐day‐old piglets were processed for 10× scRNA‐seq analysis. A total of 14 358 cells (5791 cells from LD and 8567 cells from SOL) passed quality control. Totally, five cell types were annotated by unsupervised uniform manifold approximation and projection (UMAP) analysis, including myo‐lineage cells (with expression of PAX7, CD82,^S21^ MYMK,^10^ ITGA7 and CDKN1C), FAPs (PDGFRA, PDGFRB, FGF7 and TIMP3), endothelial cells (CDH5^S22^), tenocytes (COMP^S22^) and immune cells (C1QA^S22^), and all of these cell types were detected in both LD and SOL samples (*Figure* *1* *A*–*C*). Gene Ontology (GO) terms enriched in myo‐lineage cells were related to the generation of precursor metabolites, energy and muscle organ development processes (*Figure* *S1* *A*). FAPs were characterized with GO terms involved in blood vessel morphogenesis (*Figure* *S1* *B*). Interestingly, the proportion of myo‐lineage cells was higher in LD, while FAPs were more abundant in SOL (*Figure* *1* *D*).

### Porcine myogenic cells were heterogeneous populations

**Figure 2:**
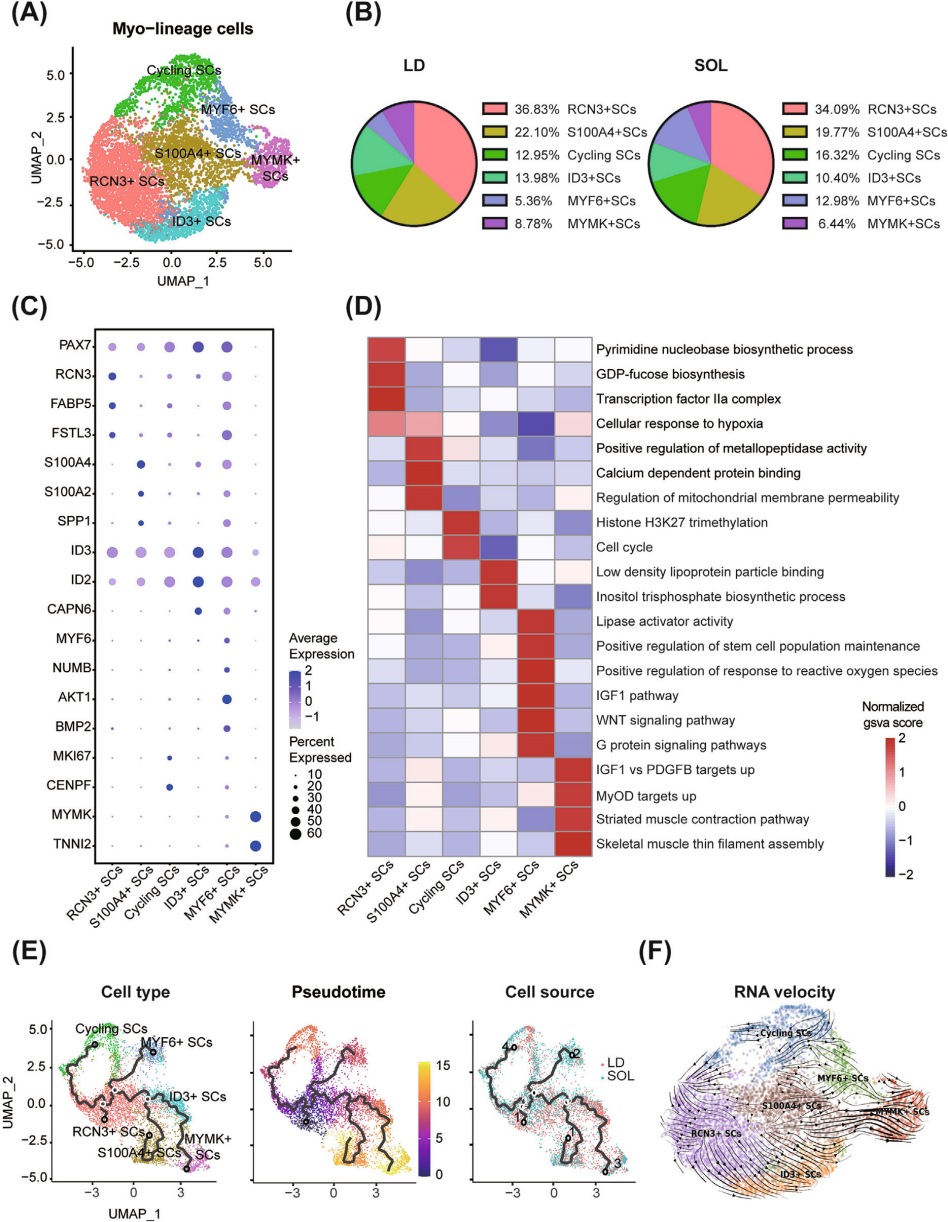
Porcine myogenic cells were heterogeneous populations. (A) UMAP plot of porcine myo‐lineage cell subpopulations. (B) Pie graphs of the composition of myo‐lineage cell subtypes in LD and SOL. (C) Dot plots showing differently expressed marker genes associated with porcine myo‐lineage cell subpopulations. (D) Heatmap of pathway enrichment score for different porcine myo‐lineage cell subpopulations analysed by GSVA. The colour showed the normalized gsva score of each pathway. (E) Pseudotime trajectory inferred across all porcine myo‐lineage cells analysed by Monocle 3. Overlaid colours indicate identified myo‐lineage cells subpopulations, pseudotime and cell sources. (F) RNA velocity analysis of six myo‐lineage cell subpopulations.

Using unsupervised UMAP analysis, myo‐lineage cells were further classified into six subpopulations, including RCN3^+^ SCs, S100A4^+^ SCs, ID3^+^ SCs, cycling SCs (MKI67^+^),^11^ MYF6^+^ SCs and MYMK^+^ SCs^10^ (*Figure* *2* *A*), and the proportions of cycling and MYF6^+^ SCs were relatively higher in the SOL group (*Figure* *2* *B*). The markers for each subpopulation were shown in *Figures* *2* *C* and *S2A*. Considering RCN3,^S23^ FSTL3^S23^ and S100A4^S24^ ^,^ ^S25^ were identified as potential markers for mesenchymal stem cells (MSCs), RCN3^+^ and S100A4^+^ SCs might be MSC‐like subsets. GSVA showed that RCN3^+^ SCs were characterized with pyrimidine and GDP‐fucose biosynthesis processes, while S100A4^+^ SCs were enriched with GO terms involved in calcium‐dependent protein binding and mitochondrial membrane permeability pathways (*Figure* *2* *D*). ID3 and ID2 were direct targets of PAX7 in quiescent MuSCs^12^; thus, ID3^+^ SCs were quiescent SCs (qSCs) with a high level of PAX7^13^ (*Figures* *2* *C* and *S2A*). MYF6^+^ SCs possessed higher expression of NUMB,^S26^ a marker of asymmetrical division and self‐renewal, and were enriched with GO terms related to stem cell population maintenance (*Figure* *2* *C*,*D*). MYMK^+^ SCs expressed low levels of PAX7 and MYOG, indicating that it might be a subpopulation committed to myogenic differentiation (*Figures* *2* *C*,*D* and *S2A*,*B*).

In porcine myo‐lineage cells, AUCell gene set score analysis was performed to assess the enrichment score of gene signatures of qSCs and activated SCs (aSCs) in mice^13^ and human.^S27^ MYF6^+^ SCs and cycling SCs exhibited the highest enrichment score with mice‐activated T3‐SC (*Figure* *S2C*). Meanwhile, genes enriched in human qSC were also found in porcine ID3^+^ SCs, and the gene signatures of human qSC, aSC and differentiated myoblast (diff mb) were enriched in porcine MYF6^+^ SCs (*Figure* *S2D*).

Pseudotime and RNA velocity analyses further revealed the complex developmental trajectories within heterogeneous SC populations in pigs. RCN3^+^ SCs were the origin of the pseudotime trajectory and committed to ID3^+^ and cycling SCs (*Figure* *2* *E*,*F*). Cycling SCs are also committed to RCN3^+^ SCs. Most of the S100A4^+^ SCs were committing towards MYMK^+^ SCs (*Figure* *2* *E*,*F*).

Co‐expression analysis identified modules enriched in RCN3^+^ SCs (Modules 9 and 5), S100A4^+^ SCs (Modules 1 and 6), ID3^+^ SCs (Module 8) and MYF6^+^ SCs (Modules 2 and 3) (*Figure* *S3A*,*B*). GO terms related to RNA processing, cellular respiration, extracellular matrix organization and protein catabolic process were enriched in RCN3^+^, S100A4^+^, ID3^+^ and MYF6^+^ SCs, respectively (*Figure* *S3C*).

There were 711 differentially expressed genes between LD and SOL SCs (248 up‐regulated in LD and 463 up‐regulated in SOL, *Figure* *S4A*). As gene set enrichment analysis (GSEA) indicated, myo‐lineage cells derived from SOL were enriched with cell cycle and FGFR signalling pathways, while those of LD exhibited higher enrichment scores of myogenesis and oxidative phosphorylation (*Figure* *S4B*). The proliferative potential of MuSCs in fast LD (95.05 ± 0.26% fast myofibres and 4.95 ± 0.26% slow myofibres, *Figure* *S5A*) and slow SOL (71.31 ± 5.45% fast myofibres and 28.69 ± 5.45% slow myofibres, *Figure* *S5A*) was further estimated both in vivo and in vitro. SOL muscle possessed a higher proportion of Pax7^+^ cells (21.66 ± 3.66% in SOL vs. 12.03 ± 1.78% in LD, *P* = 0.009) and Ki67^+^/Pax7^+^ cells (12.04 ± 1.75% in SOL vs. 6.18 ± 1.07% in LD, *P* = 0.029) (*Figure* *S5A*). MuSCs derived from LD (LD‐MuSCs) and SOL (SOL‐MuSCs) were isolated, purified (>98% Pax7^+^MyoD^+^ cells) and cultured simultaneously to compare their proliferation potential (*Figure* *S5B*). Two days after seeding, SOL‐MuSCs possessed a higher proportion of EdU^+^ cells (55.45 ± 1.74% in SOL‐MuSCs vs. 48.80 ± 1.33% in LD‐MuSCs, *P* = 0.009) and Ki67^+^ cells (23.57 ± 2.74% in SOL‐MuSCs vs. 11.40 ± 1.69% in LD‐MuSCs, *P* = 0.002) (*Figure* *S5C*). Compared with LD‐MuSCs, SOL‐MuSCs possessed higher expression of proliferation markers, such as more cyclin D (+72.16 ± 25.15%, *P* = 0.045), more proliferating cell nuclear antigen (PCNA) (+132.9 ± 33.4%, *P* = 0.028) and less p21 protein level (−50.76 ± 13.94%, *P* = 0.022), with no significant difference in Ki67 and Pax7 proteins (*Figure* *S5D*). In summary, myogenic cells derived from SOL exhibited better potential of proliferation than those from LD.

### FGF7 mediated the interaction between fibro‐adipogenic progenitors and myogenic cells

**Figure 3:**
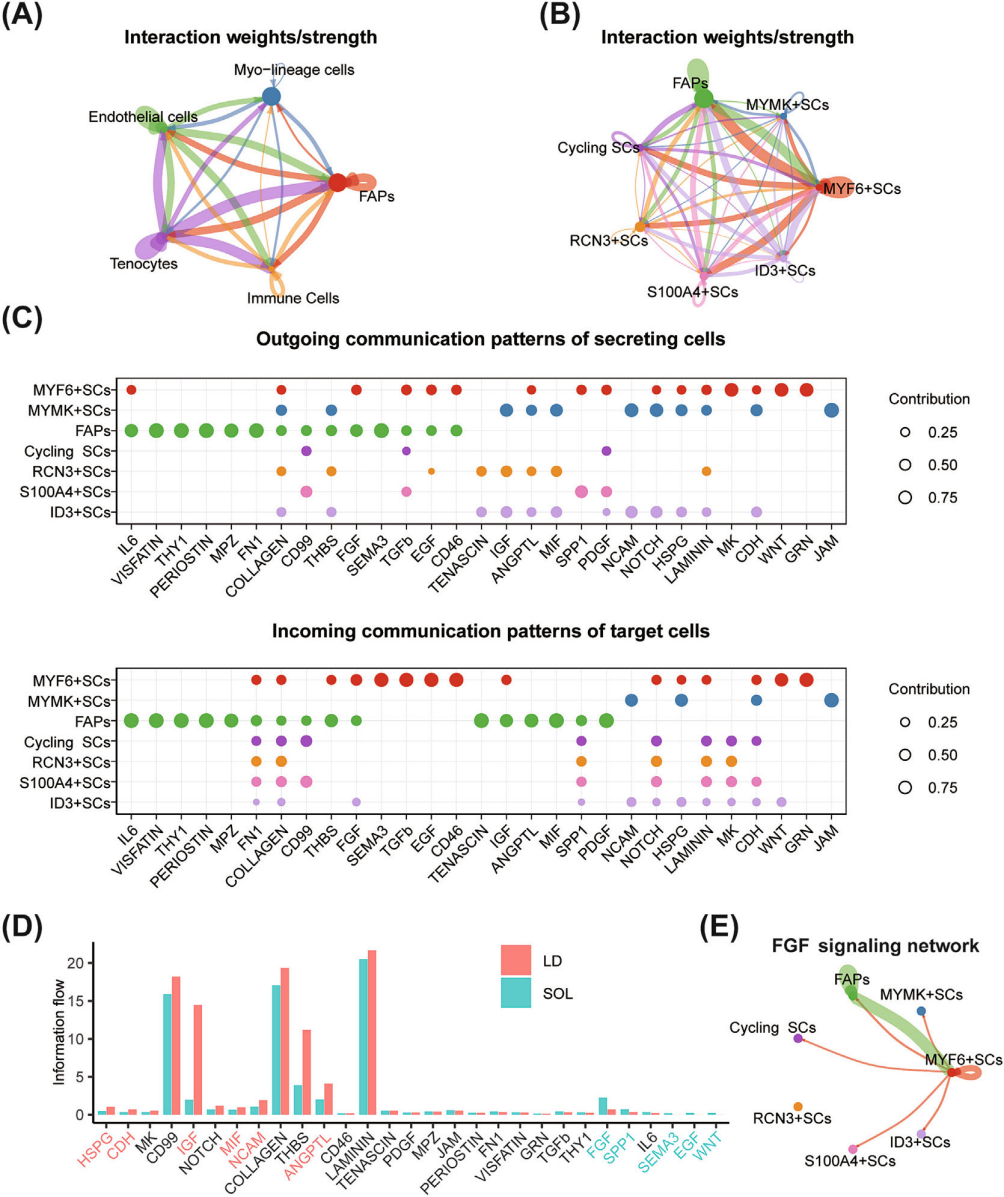
Cell–cell interactions across porcine FAPs and myogenic cell subpopulations. (A) Interaction weights/strength across all cell types in porcine muscle mononuclear cells. (B) Interaction weights/strength across FAPs and myo‐lineage cell subpopulations. (C) The dot plots showing outgoing communication patterns of secreting cells and incoming communication patterns of target cells across FAPs and myo‐lineage cell subpopulations. The dot size is proportional to the contribution score computed from pattern recognition analysis. A higher contribution score implies that the signalling pathway is more enriched in the corresponding cell group. (D) All signalling pathways were ranked based on their differences in overall information flow within the inferred networks between LD and SOL. A paired Wilcoxon test was performed to determine whether there were significant differences between the two datasets. The left signalling pathways coloured red were more enriched (*P* < 0.05) in LD, the middle pathways coloured black were equally enriched in LD and SOL, and the right ones coloured green were more enriched (*P* < 0.05) in SOL. (E) The inferred FGF signalling network across FAPs and myo‐lineage cell subsets, and the edge width represented the communication probability.

Cell–cell communication analysis using CellChat figured out various intercellular interactions across all cell types (*Figure* *3* *A*). FAPs accounted for the largest population in porcine muscle mononuclear cells apart from myo‐lineage cells (*Figure* *1* *A*) and were enriched with genes associated with the organism interaction pathway (*Figure* *S1* *B*). Therefore, cell interactions across FAPs and myo‐lineage cell subgroups were analysed specifically. FAPs showed strong interaction strength with myo‐lineage cells, especially with MYF6^+^ SC (*Figure* *3* *B*). Pattern recognition analysis uncovered a total of 29 signalling pathways (*Figure* *3* *C*), which could be divided into four outgoing and four incoming communication patterns (*Figure* *S6*). Pathways in Outgoing Patterns 1 and 4 (including NOTCH, IGF, NCAM, CDH, JAM, PDGF, ANGPTL and SPP1) were mainly secreted by RCN3^+^, ID3^+^, S100A4^+^, Cycling and MYMK^+^ SC, where IGF, MIF, PDGF, ANGPTL and SPP1 could target FAPs (*Figure* *S6*). Pathways in Outgoing Pattern 2 (including FN1, MPZ, FGF, THY1, IL6, PERIOSTEN, VISFATIN and SEMA3) were mainly secreted by FAPs, where FGF and SEMA3 could target MYF6^+^ SC (*Figure* *S6*). Outgoing Pattern 3 was mainly composed of myogenic secretion and target signalling pathways, including MK, WNT and GRN derived from MYF6^+^ SC (*Figure* *S6*). The communication probability of HAPG, CDH, IGF, MIF, NCAM and ANGPTL pathways was significantly stronger in LD, as indicated by information flow (red in *Figure* *3* *D*). There were five pathways (FGF, SPP1, SEMA3, EGF and WNT) significantly stronger in SOL (green in *Figure* *3* *D*), and the FGF pathway was primarily derived from FAPs and secreted towards MYF6^+^ SC (*Figure* *3* *E*).

**Figure 4:**
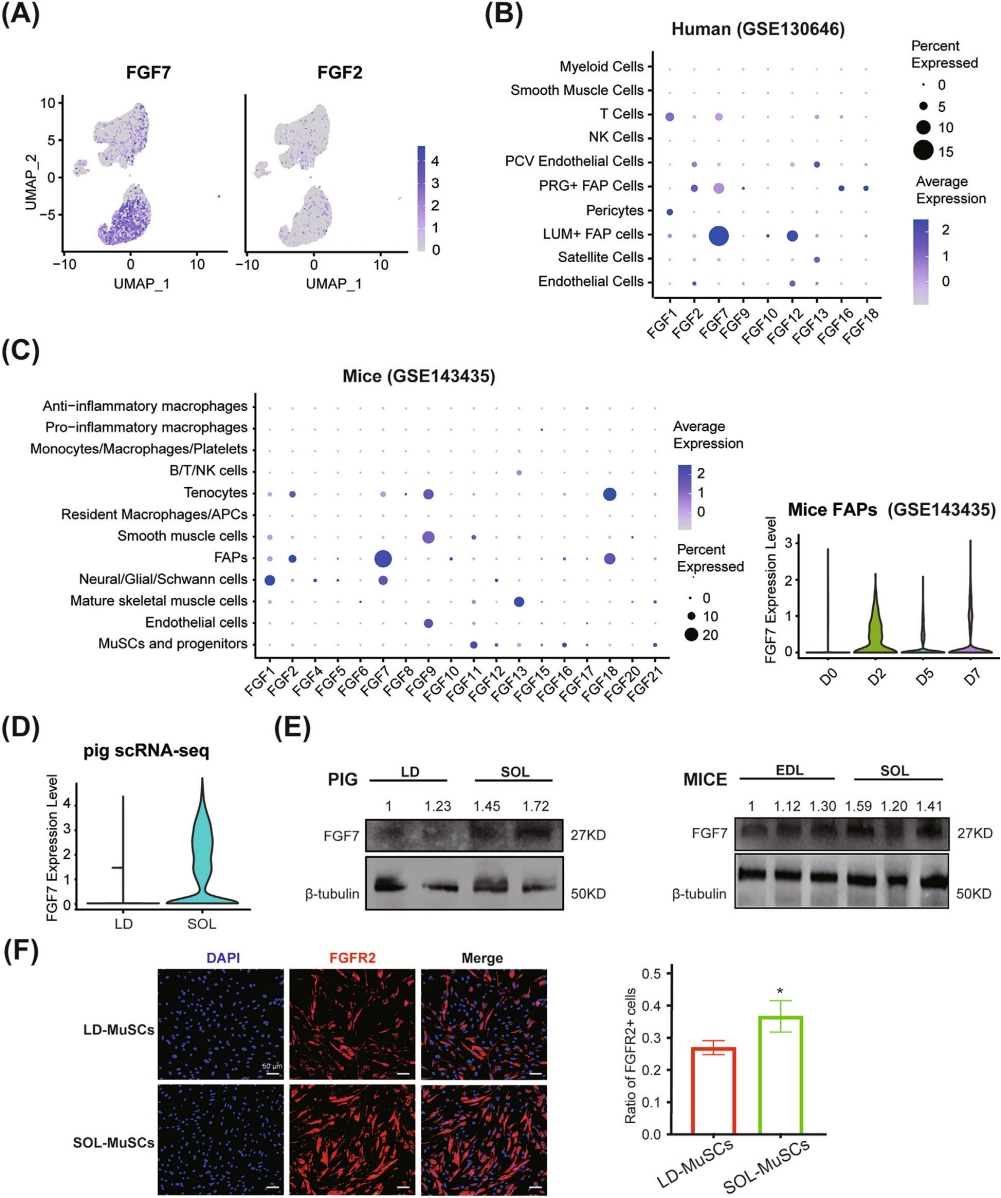
FGF7 mediated the interaction between FAPs and myogenic cells. (A) UMAP plots of FGF7 and FGF2 expression in porcine muscle mononuclear cells. (B) Dot plot of FGF members in human muscle scRNA‐seq data published by Rubenstein et al.^14^ The dot size was proportional to the per cent of FGF expression in the corresponding cell group. And the colour showed the average expression of FGFs. (C) Dot plot of FGF members in mice skeletal muscle scRNA‐seq data, as well as the violin plot of FGF7 expression in mice FAPs collected at 0, 2, 5 and 7 days after injury published by De Micheli et al.^15^ (D) Violin plot of FGF7 between LD and SOL mononuclear cells. (E) Western blot analyses of FGF7 in LD and SOL muscles in 3‐day‐old piglets and EDL and SOL muscles in 8‐month‐old mice. The quantification of FGF7 protein levels was normalized to both β‐tubulin and the first sample on the left. (F) Immunofluorescence staining of FGFR2 in LD‐MuSCs and SOL‐MuSCs, and the ratio of FGFR2^+^ cells from LD‐MuSCs and SOL‐MuSCs were quantified. The scale bar is 50 μm (*n* = 3). Data are presented as mean ± SD (*P < 0.05).

Notably, FGF7 was the most highly expressed FGF and mainly enriched in FAPs in our study (*Figure* *4* *A*), as well as in human^16^ (*Figure* *4* *B*) and mouse^17^ (*Figure* *4* *C*) scRNA‐seq data. The expression of FGF7 in FAPs of mice TA muscles reached a peak 2 days after injury (*Figure* *4* *A*–*C*). Besides, a stronger FGF7 signal in slow SOL than those in fast LD was detected by porcine scRNA‐seq (*Figure* *4* *D*), more FGF7 in SOL than LD of newborn piglets was further conformed with western blot, and similar results were also observed in 8‐month‐old mice (*Figure* *4* *E*).

Considering FGFR2 was the receptor with the highest affinity with FGF7,^16^ the expression of FGFR2 in myogenic cells was detected. In porcine scRNA‐seq data, FGFR2 was mostly enriched in S100A4^+^, cycling and MYMK^+^ SCs (*Figure* *S7A*). The mRNA levels of FGFR2 were increased during the proliferation of C2C12 (*Figure* *S7B*) and porcine MuSCs (~8.2 times higher in 48 h than 0 h, *Figure* *S7C*) and were significantly higher in SOL‐MuSCs than LD‐MuSCs (*Figure* *S7D*). Immunofluorescence (IF) staining of proliferating MuSCs showed that LD‐MuSCs and SOL‐MuSCs possessed 26.94 ± 2.15% and 36.68 ± 4.85% FGFR2^+^ cells, respectively (*Figure* *4* *F*). The AUCell assay showed that the gene set of FGF‐activated receptor activity was especially enriched in MYF6^+^ and ID3^+^ SCs, and the type 2 FGFR binding gene set was significantly enriched in MYF6^+^ and RCN3^+^ subpopulations (*Figure* *S7E*).

### FGF7 promoted proliferation of myogenic cells via FGFR2

**Figure 5:**
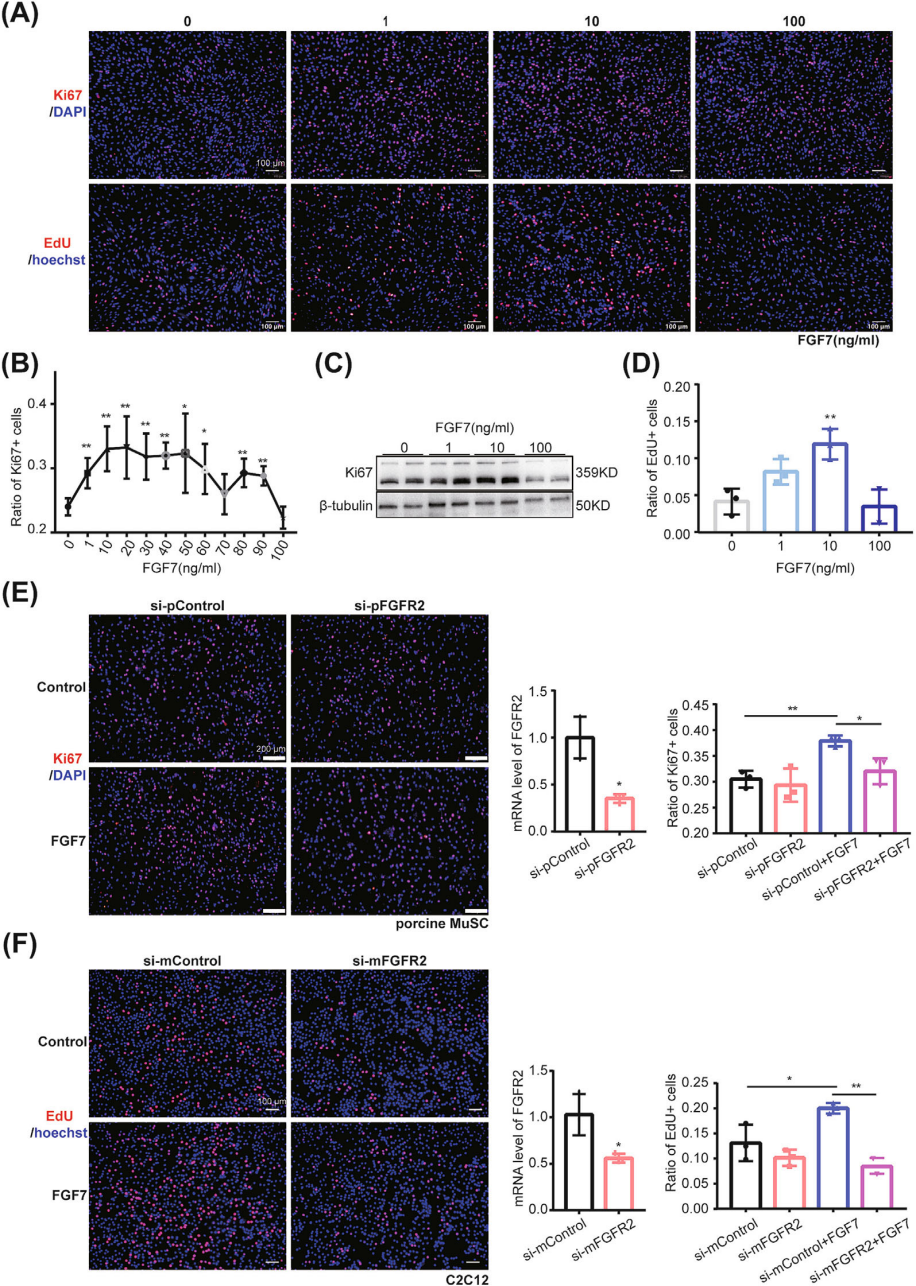
FGF7–FGFR2 promoted proliferation of myogenic cells. (A) Immunofluorescence staining with EdU (red) and Hoechst (blue), Ki67 (red) and DAPI (blue) in porcine MuSCs treated with a series of concentrations of FGF7 recombinant protein (0, 1, 10 and 100 ng/mL). The scale bar is 100 μm. (B) Quantification of the proportion of Ki67^+^ cells in porcine MuSCs treated with a series of concentrations of FGF7 recombinant protein (0, 1, 10, 20, 30, 40, 50, 60, 70, 80, 90 and 100 ng/mL) (*n* = 3). (C) Western bolt analysis of Ki67 in porcine MuSCs treated with FGF7 recombinant protein (0, 1, 10 and 100 ng/mL). (D) Quantification of the proportion of EdU^+^ cells in porcine MuSCs treated with FGF7 recombinant protein (0, 1, 10 and 100 ng/mL) (*n* = 3). (E) Immunofluorescence staining with Ki67 (red) and DAPI (blue) in porcine MuSCs treated with si‐pFGFR2 and 10‐ng/mL FGF7 recombinant protein, and the mRNA level of porcine FGFR2 was detected to show its knockdown efficiency, and the ratio of Ki67^+^ cells was quantified. The scale bar is 200 μm (*n* = 3). (F) Immunofluorescence staining with EdU (red) and Hoechst (blue) in C2C12 cell line treated with si‐mFGFR2 and 10‐ng/mL FGF7 recombinant protein, and the mRNA level of mice FGFR2 was detected to show its knockdown efficiency, and the ratio of EdU^+^ cells was quantified. The scale bar is 100 μm (*n* = 3) (*P < 0.05 and ^**^ *P* < 0.01). Data are presented as mean ± SD.

The distinct expression pattern of FGF7–FGFR2 in LD‐MuSCs and SOL‐MuSCs and the selective targeting of FGF7 on proliferating and aSCs encouraged us to test the function of FGF7 on cell proliferation. A series of concentrations of FGF7 recombinant protein (0, 1, 10, 20, 30, 40, 50, 60, 70, 80, 90 and 100 ng/mL) were administered to proliferating porcine MuSCs (*Figure* *5* *A*). FGF7 significantly expanded the proportion of Ki67^+^ cells and increased Ki67 protein levels in a dose‐dependent manner, entering a plateau phase when the dose exceeded 10 ng/mL and losing its effect when the dose reached 100 ng/mL (*Figure* *5* *A*–*C*). EdU staining also showed that 10‐μg/mL FGF7 presented the best promoting effect (4.03 ± 0.81%, *P* = 0.008) on porcine MuSC proliferation (*Figure* *5* *A*,*D*). Si‐pFGFR2 effectively knocked down FGFR2 on porcine MuSCs by 65.81 ± 13.26% (*P* = 0.038) and significantly blocked the improved Ki67^+^ population induced by FGF7 (*Figure* *5* *E*). Knockdown of FGFR2 in C2C12 also significantly (11.5 ± 1.08%, *P* = 0.004) blocked the elevated EdU^+^ population (6.87 ± 2.17%, *P* = 0.034) upon FGF7 treatment (*Figure* *5* *F*). FGF7 significantly increased phosphorylation of p38 mitogen‐activated protein kinase (MAPK) and extracellular signal‐regulated kinase (ERK) in proliferating C2C12, while si‐FGFR2 greatly blocked these effects (*Figure* *S7F*).

### FGF7 promoted *tibialis anterior* muscle regeneration after injury in mice

**Figure 6:**
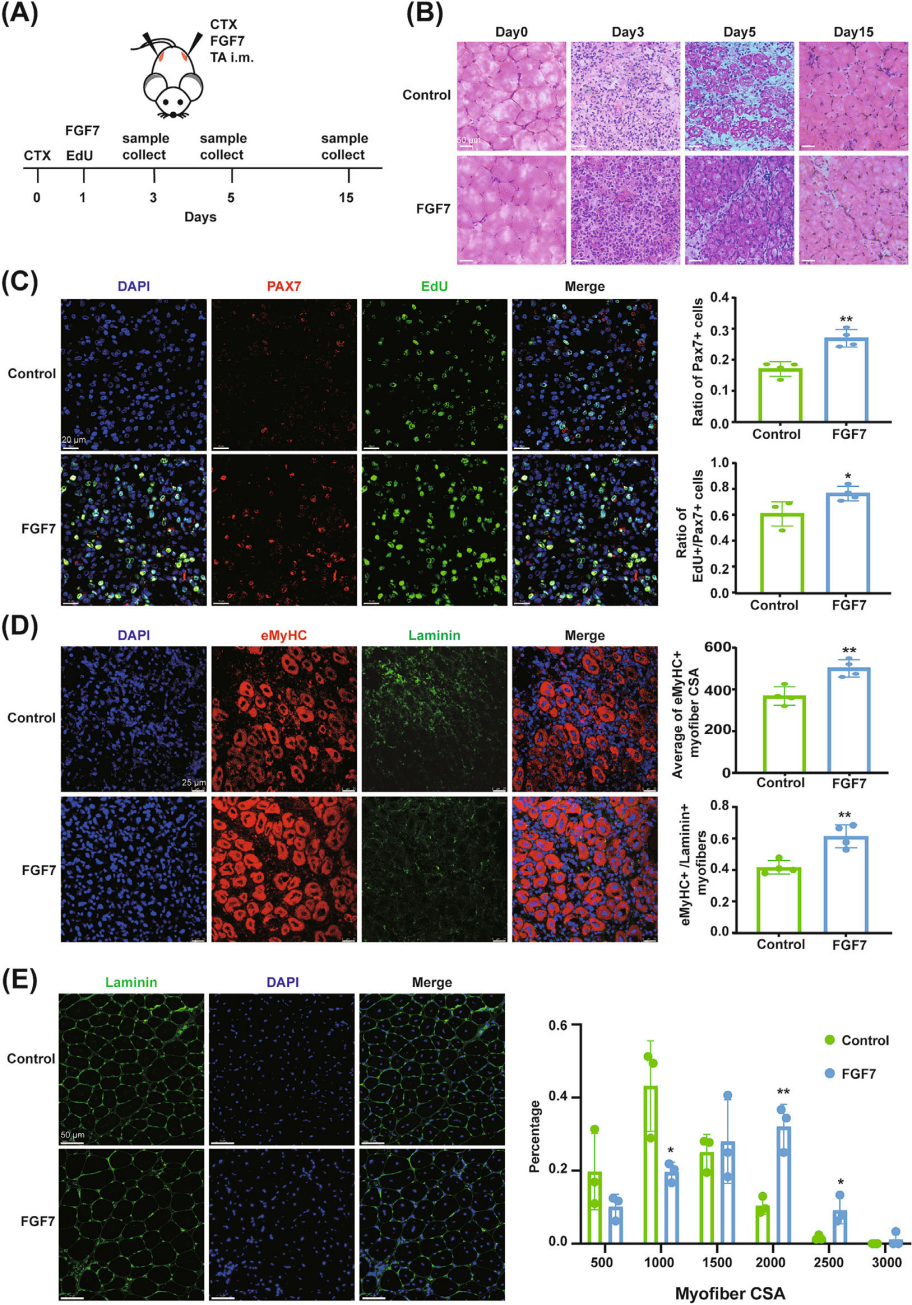
FGF7 promoted muscle regeneration after injury in mice. (A) Experimental schematic. The TA muscles of 8‐week‐old mice were infected with CTX at 0 days and FGF7 recombinant protein (control group was infected with 0.9% NaCl) at 1 day, and samples were collected at 0, 3, 5 and 15 days. EdU was injected intraperitoneally for two consecutive days before analysis. (B) H&E staining of TA muscle at 0, 3, 5 and 15 days after injury (scale bar: 50 μm) (*n* = 5). (C) Immunofluorescence staining with EdU (green), Pax7 (red) and DAPI (blue) of TA muscle at 3 days after injury, and the ratio of Pax7^+^ cells and EdU^+^ versus Pax7^+^ cells was quantified. The scale bar is 20 μm (*n* = 4). (D) Immunofluorescence staining with eMyHC (red), laminin (green) and DAPI (blue) of TA muscle at 5 days after injury, and the average cross‐sectional area (CSA) and ratio of eMyHC^+^ myofibre (vs. laminin^+^ myofibres) were quantified. The scale bar is 25 μm (*n* = 4). (E) Immunofluorescence staining with laminin (green) and DAPI (blue) of TA muscle at 15 days after injury, and the myofibre CSA was measured with ImageJ. The scale bar is 50 μm (*n* = 3) (*P < 0.05 and ^**^ *P* < 0.01). Data are presented as mean ± SD.

A TA injury model was established to explore the effect of FGF7 on MuSC proliferation during muscle regeneration (*Figure* *6* *A*). H&E staining showed that after CTX injury, FGF7‐treated mice exhibited less fibrosis and more myofibres with centralized nucleus at 3 and 5 days, respectively (*Figure* *6* *B*). At 3 days after injury, FGF7 significantly expanded the proportion of Pax7^+^ cells by 9.82 ± 1.84% (*P* = 0.002) and EdU^+^/Pax7^+^ cells by 15.68 ± 5.45% (*P* = 0.028) (*Figure* *6* *C*). At 5 days after injury, FGF7 improved the average CSA and number of eMyHC^+^ myofibres by 132.7 ± 30.25 μm^2^ (*P* = 0.005) and 19.7 ± 4.25% (*P* = 0.004), respectively (*Figure* *6* *D*). At 15 days after injury, laminin staining showed that there was a significantly higher proportion of myofibres in the 1500‐ to 2000‐μm^2^ (*P* < 0.01) and 2000‐ to 2500‐μm^2^ ranges (*P* < 0.05) and a smaller proportion in the 500‐ to 1000‐μm^2^ range (*P* < 0.05) in FGF7‐treated mice (*Figure* *6* *E*).

### FGF7 delayed myogenic cell senescence in vitro and in vivo

**Figure 7:**
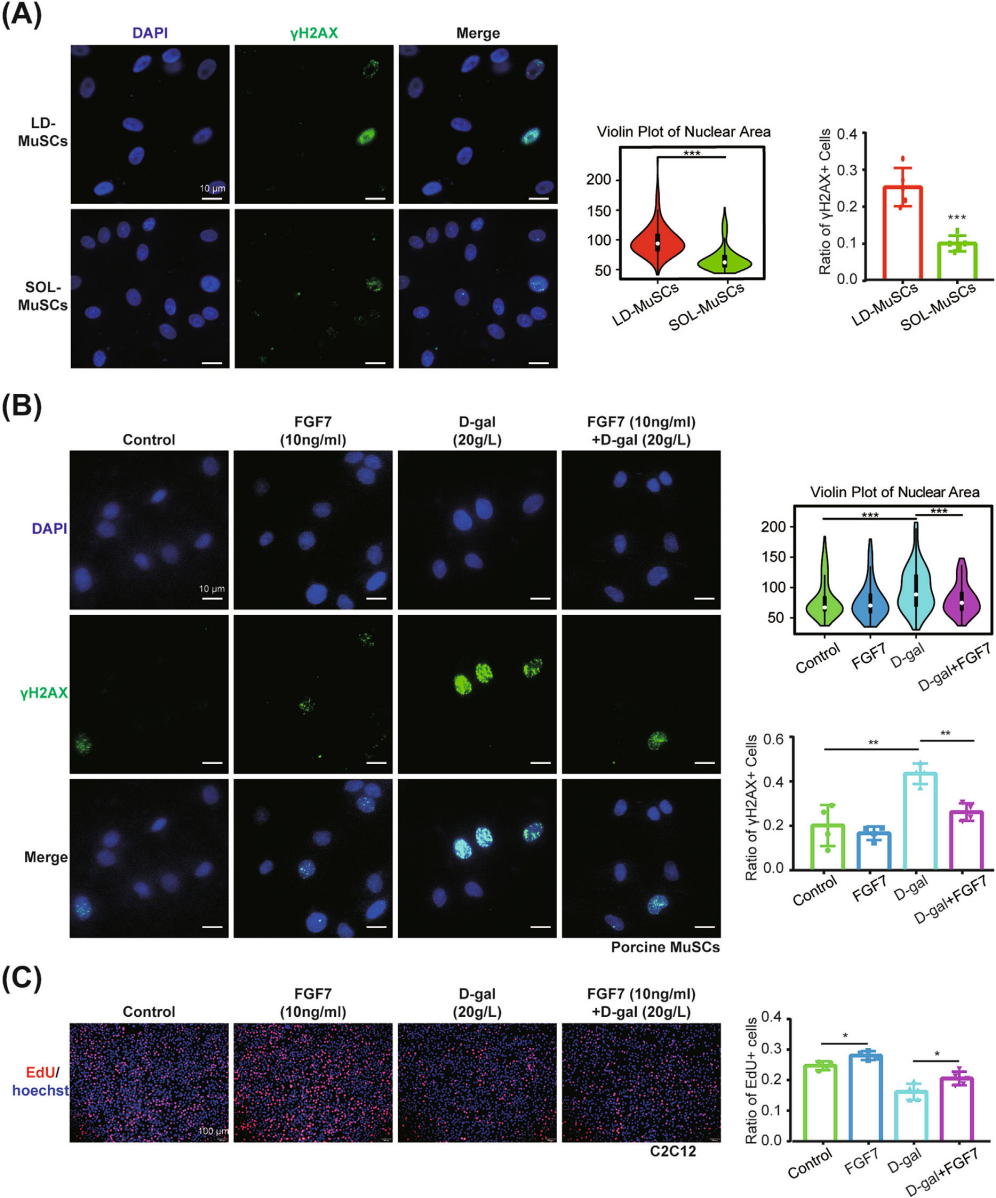
FGF7 delayed myogenic cell senescence. (A) Immunofluorescence staining with γH2AX (green) and DAPI (blue) was detected in synchronously cultured passage 15 LD‐MuSCs and SOL‐MuSCs, and the nuclear area (*n* = 200 nuclei) and ratio of γH2AX^+^ cells (*n* = 5 samples) were quantified. The scale bar is 10 μm. (B) Immunofluorescence staining with γH2AX (green) and DAPI (blue) was detected in porcine MuSCs treated with 10‐ng/mL FGF7 recombinant protein, 20‐g/L d‐gal and 20‐g/L d‐gal + 10‐ng/mL FGF7, and the nuclear area (*n* = 200 nuclei) and ratio of γH2AX^+^ cells (*n* = 4 samples) were quantified. The scale bar is 10 μm. (C) Immunofluorescence staining with EdU (red) and Hoechst (blue) was detected in C2C12 treated with 10‐ng/mL FGF7 recombinant protein, 20‐g/L d‐gal and 20‐g/L d‐gal + 10‐ng/mL FGF7, and the ratio of EdU^+^ cells was quantified. The scale bar is 100 μm (*n* = 5) (*P < 0.05, ^**^ *P* < 0.01 and ^***^ *P* ≤ 0.001). Data are presented as mean ± SD.

LD‐MuSCs and SOL‐MuSCs were cultured synchronously until passage 15, and IF was stained with γH2AX and DAPI (*Figure* *7* *A*). SOL‐MuSCs exhibited significantly (*P* ≤ 0.001) smaller size of nuclei and much lower percentage of γH2AX^+^ cells (10.21 ± 2.15%) than LD‐MuSCs (25.54 ± 5.20%) (*Figure* *7* *A*), indicating that SOL‐MuSCs were more resistant to replication senescence.

To test the effects of FGF7 on cell senescence, 20‐g/L d‐gal was used to induce severe senescence in porcine MuSCs (*Figure* *S8A*–*C*), and 10‐μg/mL recombinant FGF7 effectively (*P* < 0.001) mitigated the enlarged nuclei size caused by d‐gal (*Figure* *7* *B*). The proportion of γH2AX^+^ cells was increased by 23.2 ± 5.16% (*P* = 0.004) in d‐gal‐treated porcine MuSCs, which was reduced by FGF7 by 17.19 ± 3.05% (*P* = 0.001) (*Figure* *7* *B*). Similarly, 20‐g/L d‐gal was used to induce senescence in C2C12,^S15^ which significantly expanded the nuclei size, while FGF7 effectively (*P* < 0.001) rescued it (*Figure* *S8D*). Consistently, d‐gal dramatically increased the proportion of γH2AX^+^ cells by 26.2 ± 1.26% (*P* < 0.001) in C2C12, while FGF7 reduced that by 13.77 ± 2.90% (*P* = 0.009) (*Figure* *S8D*). FGF7 even reduced the basal proportion of γH2AX^+^ cells by 5.05 ± 1.26% (*P* = 0.046), compared with the control without d‐gal (*Figure* *S8D*). d‐gal reduced the proportion of EdU‐positive cells by 8.53 ± 1.48% (*P* < 0.001) in C2C12, while FGF7 rescued it by 4.34 ± 1.54% (*P* = 0.023) (*Figure* *7* *C*).

**Figure 8:**
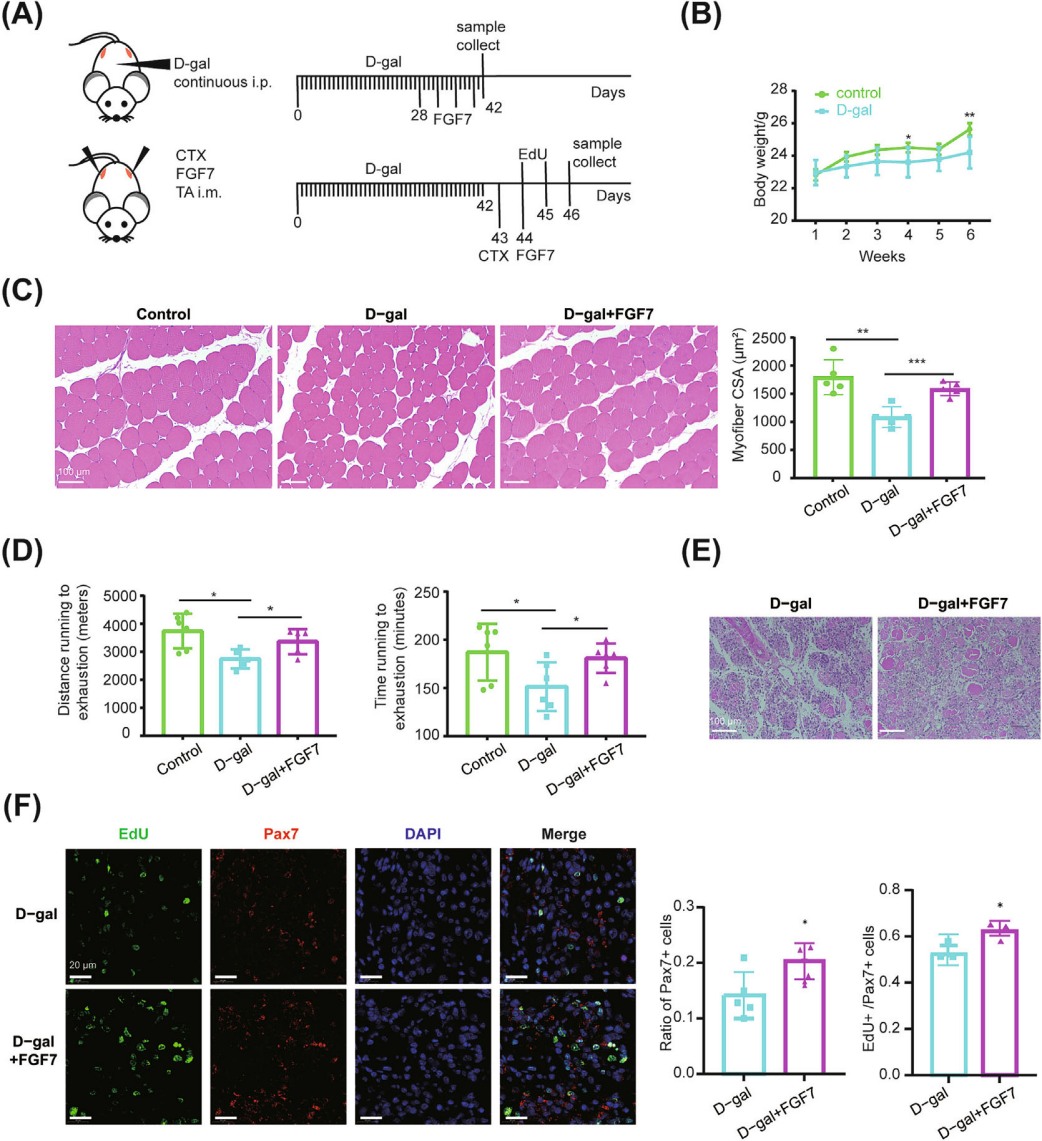
FGF7 delayed d‐gal‐induced muscle ageing in mice. (A) Experimental schematic. The 8‐week‐old mice were treated with continuous intraperitoneal (i.p.) injection of d‐gal for 6 weeks. During the last 2 weeks, twice‐weekly TA intramuscular (i.m.) injections of FGF7 were performed. Mice running experiments were performed after three injections of FGF7. And samples for H&E staining were collected 1 day after the fourth injection of FGF7. Following successful d‐gal‐induced ageing, TA was injected with CTX. FGF7 injection was performed 1 day later, and EdU was injected intraperitoneally for two consecutive days before analysis. Samples for H&E staining and immunofluorescence staining were collected 3 days after CTX injection. (B) Body weight changes among 6 weeks of d‐gal i.p. injection (*n* = 6). (C) H&E staining of TA muscle after four injections of FGF7, and the myofibre CSA was analysed with ImageJ. The scale bar is 100 μm (*n* = 5). (D) Distance running to exhaustion (m) and time running to exhaustion (min) (*n* = 6). (E) H&E staining of TA muscle at 3 days after injury (scale bar: 20 μm) (*n* = 5). (F) Immunofluorescence staining with EdU (green), Pax7 (red) and DAPI (blue) of TA muscle at 3 days after injury, and the ratio of Pax7^+^ cells and EdU^+^ versus Pax7^+^ cells was quantified. The scale bar is 20 μm (*n* = 5) (*P < 0.05, ^**^ *P* < 0.01 and ^***^ *P* ≤ 0.001). Data are presented as mean ± SD.

In the d‐gal‐induced ageing model (*Figure* *8* *A*), mice body weight was significantly decreased by 1.423 ± 0.468 g (*P* = 0.006) (*Figure* *8* *B*), TA myofibre CSA accordingly became smaller (*P* < 0.01), and FGF7 effectively (*P* < 0.001) rescued the loss of myofibre size (*Figure* *8* *C*). d‐gal‐treated mice also exhibited damaged running ability, as indicated by a rough reduction in running distance by 26.6% (*P* = 0.011) and 19.1% (*P* = 0.048) in time running to exhaustion (*Figure* *8* *D*). FGF7 significantly improved the running performance of d‐gal mice, as indicated by the great increase in distance and time running to exhaustion by 22.4% (*P* = 0.040) and 19.5% (*P* = 0.034), respectively (*Figure* *8* *D*).

CTX was sequentially used to induce TA injury in d‐gal‐treated mice (*Figure* *8* *A*). H&E staining showed that FGF7‐injected mice exhibited less inflammatory infiltration at 3 days after injury (*Figure* *8* *E*). Meanwhile, FGF7‐treated muscle possessed a significantly higher proportion of Pax7^+^ cells (*P* = 0.020) and EdU^+^/Pax7^+^ cells (*P* = 0.031) at 3 days after injury (*Figure* *8* *F*).

## Discussion

MuSCs exert critical roles during skeletal muscle regeneration, ageing, exercise and other adaptive changes.^17^ MuSC is a heterogeneous population with cells at different stages, such as quiescence, activation, proliferation and differentiation,^18^ and each can be distinguished by distinct marker genes, such as PAX7, MYF5, MYOD and MYOG, respectively.^19^, ^20^, ^21^

In our study, porcine myogenic cells were further identified as six subpopulations, including two MSC‐like SCs (RCN3^+^ and S100A4^+^), qSCs (ID3^+^), activated or self‐renewal SCs (MYF6^+^), cycling (MKI67^+^) SCs and primed to differentiation SCs (MYMK^+^). Our data were consistent with previous studies where porcine scRNA‐seq identified MYMK as a marker of differentiating SCs^10^ and ID4 (both ID4 and ID3 belong to the inhibitor of DNA binding family) as a marker of bovine qSCs.^22^ The two MSC‐like SCs were two distinct subpopulations with different cell trajectories: for RCN3^+^ SCs developed towards cycling SCs and qSCs and for S100A4^+^ SCs developed towards myogenic differentiation. Cycling SCs were also committed to RCN3^+^ SCs, showing various cell fates of MuSCs. MYF6 is generally considered to be co‐expressed with MYOD1,^S28^ and MYOD and MYF5 were induced by PAX7 upon proliferation in activated MuSCs^12^; however, low expressions of MYOD1 and MYF5 were detected in our data, as well as another porcine scRNA‐seq study,^10^ remaining a mystery deserved further investigation.

MuSC heterogeneity has been reported to be related to muscle fibre type,^23^ and our results also revealed that MuSCs derived from SOL (containing more slow fibres) possessed more cycling and MYF6^+^ SCs and relatively higher proliferative activity both in vivo and ex vivo than LD‐MuSCs (containing more fast fibres). Besides, our data suggested that SOL‐MuSCs were more resistant to replication senescence, offering an explanation why slow fibres undergo slower ageing in comparison with fast ones.^S29^

MuSCs communicate with various cells in niches, especially FAPs, during skeletal muscle regeneration and ageing.^6^, ^24^ Myogenic cell‐secreted factors could selectively target FAPs, such as PDGF signals, which could interact with PDGFR on the FAP membrane.^25^ The FAP population expands rapidly after skeletal muscle injury, generating a favourable niche for muscle regeneration.^26^ Our study revealed extensive interactions across FAPs and myogenic cell subsets, particularly MYF6^+^ SCs. Secretory factors, such as FGF,^S30^ TGFb^S31^ and WNT,^S32^ were secreted by FAPs and interacted with their receptors on myogenic cells. SEMA3 signalling, which plays an important role in skeletal muscle TW2^+^ progenitor cells,^27^ was also detected in our data.

FGF family members have been reported to play essential roles in regulating activation and proliferation of myogenic cells during skeletal muscle regeneration and ageing.^28^, ^29^ For example, FGF2 was a typical activator of MuSC proliferation as well as a key inhibitor of differentiation.^S33^ FGF7, originally known for promoting keratinocyte proliferation,^30^ has been reported to mediate the formation of myotubes in C2C12 induced by vertical vibrations.^S34^ Here, we found that FGF7 was the most highly expressed FGF member and primarily enriched in FAPs in pigs, humans^14^ and mice,^15^ as revealed by scRNA‐seq analysis. In addition, FAP‐derived FGF7 was predicted to affect proliferating or activated MYF6^+^ SCs by bioinformatic analysis, and higher expression of FGF7 was detected in SOL muscle where MuSCs presented higher proliferation activity, indicating that FGF7 might possess pro‐proliferating ability, although a scattered study speculated that FGF7 might have a modest effect on MuSC proliferation.^S35^ Here, solid experiments have shown that FGF7 effectively augmented proliferation of porcine MuSCs and C2C12, as indicated by the elevated proportion of Ki67^+^ and EdU^+^ cells.

FGFRs (FGFR1, FGFR2, FGFR3 and FGFR4) were well‐known receptors of FGFs, and their roles on myogenic cell proliferation have been wildly reported.^28^, ^31^ FGFR2, the receptor with the highest affinity for FGF7, was previously reported to exert critical roles in muscle stem cell development,^16^, ^32^ and the mRNA and protein levels of FGFR2 were positively correlated with those of MYOD in rat MuSCs.^33^ FGFR2 in muscle reached a peak at 12 h after injury and was highly expressed during C2C12 differentiation,^34^ and the circRNA sharing a common promoter with FGFR2 promoted proliferation and differentiation in chicken myoblasts.^S36^ Consistent with these reports, our work found that FGFR2 was especially expressed in the proliferating porcine MuSCs, and knockdown of FGFR2 greatly impaired the pro‐proliferating effects of FGF7 in porcine MuSCs and C2C12. We also found that MAPK/ERK signalling was activated by FGF7/FGFR2 during proliferation, which was in line with previous reports.^S37^ ^–^ ^S39^

The proliferation of MuSCs is the key process during skeletal muscle regeneration, which ensures enough myoblasts for myofibre repair.^15^, ^35^ FGF7 was found to be elevated in muscle FAPs at 2 days after injury,^15^ and disruption of FGF signalling greatly harmed MuSC activation, proliferation and muscle regeneration.^28^, ^31^ Consistently, exogenous treatment of FGF7 accelerated MuSC proliferation at 3 days after injury, increased the content of newborn myofibres and facilitated skeletal muscle regeneration in our study.

The limited proliferation capacity and cell cycle arrest are crucial features of cellular senescence, which is characterized with elevated γH2AX,^36^, ^37^ and continuous accumulation of senescent cells in skeletal muscle would lead to loss of muscle mass and muscle dysfunction.^9^, ^38^, ^39^ FGFs have been suggested to restrain cell senescence, for FGF2 could silence p21 and enhance cell proliferation in senescent MuSCs,^29^ and FGF7 partially reversed senescence of murine thymocyte progenitors via repressing of Ink4a.^S40^ In this study, d‐gal was employed to induce cell senescence both ex vivo and in vivo, and the administration of FGF7 effectively alleviated the repressed proliferation of MuSCs as well as γH2AX accumulation in MuSCs and restored the damaged motor performance induced by d‐gal, further supporting our notion that FGF7 augments MuSC proliferation both in vitro and in vivo.

In summary, our work explores the heterogeneity of myogenic cells and reveals a novel interaction between myogenic cells and FAPs mediated by FGF7–FGFR2. FGF7 promotes MuSC proliferation and delays senescence, thereby protecting against muscle injury and age‐dependent diseases.

## Conflict of interest statement

The authors declare that there are no competing interests.

## Funding

This work was supported by the National Key Research and Development Program of China (2021YFF1000602) and Shaanxi Livestock and Poultry Breeding Common Technology Research and Development Platform (2023GXJS‐02‐01).

## Supporting information


**Figure S1.** Profiling of porcine skeletal muscle cells.
**Figure S2.** Porcine myogenic cells were heterogeneous populations.
**Figure S3.** Pseudotime analysis of porcine myogenic cells.
**Figure S4.** The differently expressed genes in myo‐lineage cells derived from LD and SOL.
**Figure S5.** Satellite cells derived from porcine *soleus* muscles showed better potential of proliferation.
**Figure S6.** Cell–cell interactions between porcine FAPs and myogenic cell subpopulations.
**Figure S7.** The expression of FGFR2 in myogenic cells.
**Figure S8.** FGF7 delayed myoblast senescence.
**Table S1.** Primary antibodies used in this study.
