# Supplementary material for: Single‐cell RNA‐seq reveals novel interaction between muscle satellite cells and fibro‐adipogenic progenitors mediated with FGF7 signalling
Source: J Cachexia Sarcopenia Muscle. 2024 May 16;15(4):1388–403. doi: 10.1002/jcsm.13484 (PMC11294021; doi:10.1002/jcsm.13484)
Supplement: Supplementary file 1 — Figure S1. Profiling of porcine skeletal muscle cells. Figure S2. Porcine myogenic cells were heterogeneous populations. Figure S3. Pseudotime analysis of porcine myogenic cells. Figure S4. The differently expressed genes in myo‐lineage cells derived from LD and SOL. Figure S5. Satellite cells derived from porcine soleus muscles showed better potential of proliferation. Figure S6. Cell–cell interactions between porcine FAPs and myogenic cell subpopulations. Figure S7. The expression of FGFR2 in myogenic cells. Figure S8. FGF7 delayed myoblast senescence. Table S1. Primary antibodies used in this study. [file JCSM-15-1388-s001.doc]

**ScRNA-seq reveals novel interaction between skeletal muscle satellite cells and FAPs mediated with FGF7 signaling**

**Supplemental information includes**

**Supplemental Figures 1-8 (included in this file)**

**Supplemental Table 1 (included in this file)**

**Abbreviations**

**Supplemental Methods**

**Supplemental References**

**Figure S1 Profiling of porcine skeletal muscle cells**

(A) GSEA analysis of top 10 enriched pathways in porcine myo-lineage cells. (B) GSEA analysis of top 10 enriched pathways in porcine FAPs.

**
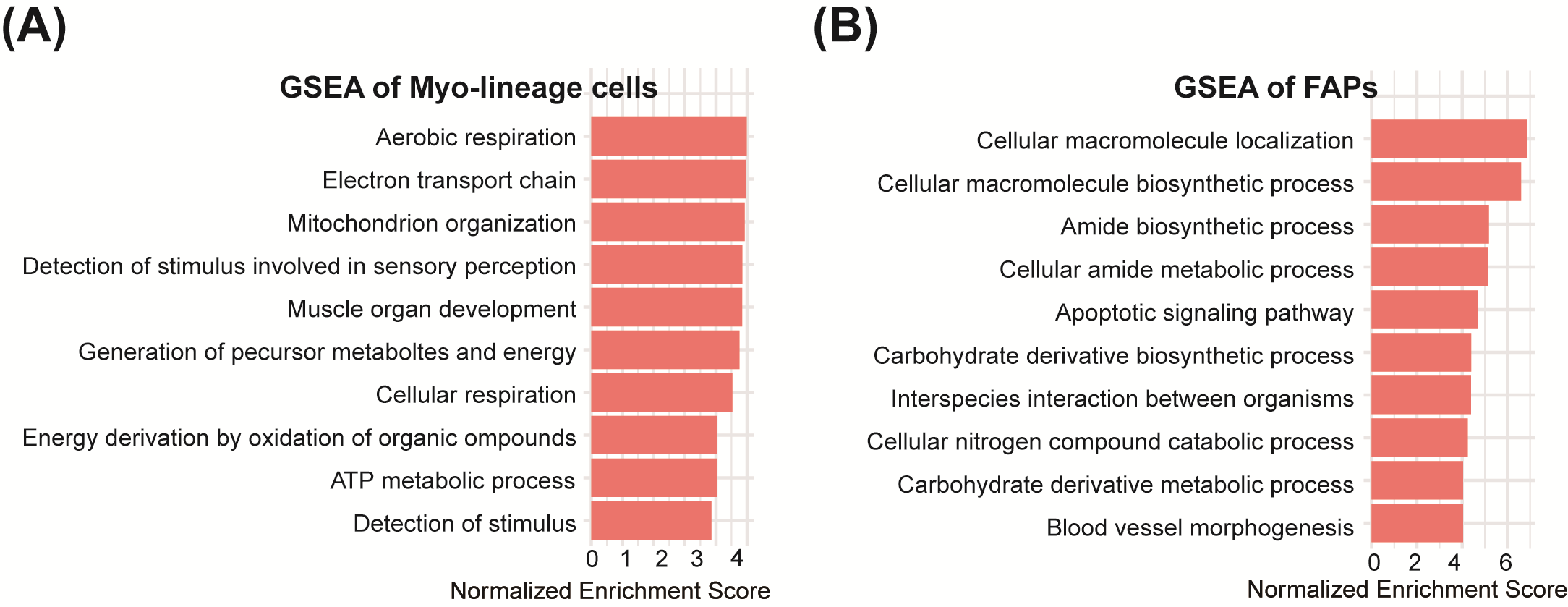
**

**Figure S2 Porcine myogenic cells were heterogeneous populations**

(A) Feature plots of markers of porcine myo-lineage cell subpopulations. (B) Violin plots of MRFs (MYF5, MYOD1, MYOG, MYF6) expression in porcine myo-lineage cell subpopulations. (C) UMAP plots of AUCell score of porcine myo-lineage cells with mice quiescent (T0-SC) and activated (T3-SC) signatures published by Machado *et al*^13^. (D) UMAP plots of AUCell score of porcine myo-lineage cells with human quiescence (qSC), activation (aSC), proliferation (pro mb) and differentiation (diff mb) signatures published by Wang *et al* **[S27]**.

**
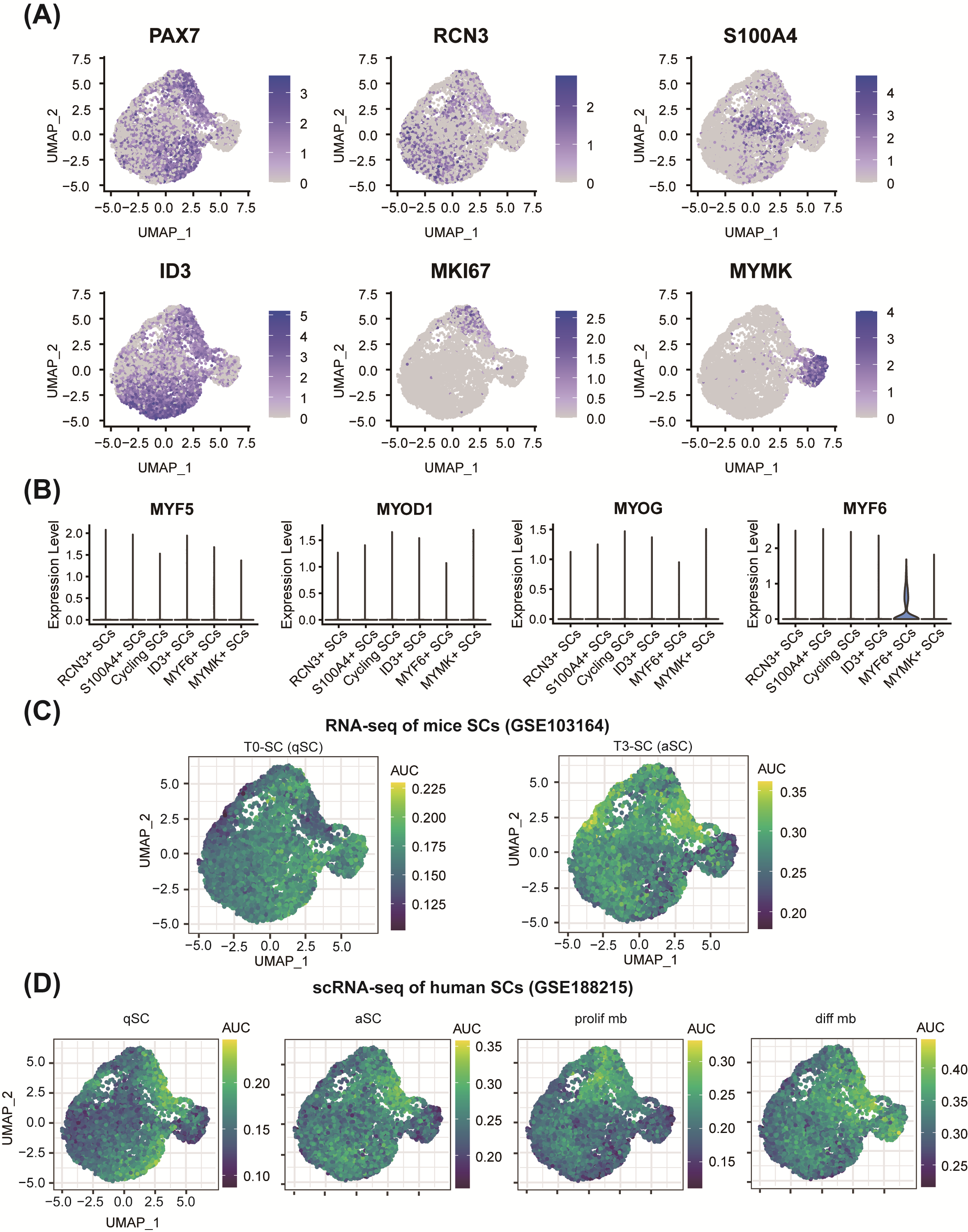
**

**Figure S3 Pseudotime analysis of porcine myogenic cells**

(A) Module analysis identified 13 modules. (B) UMAP plots showed the differential enrichment of select gene modules among myo-lineage cell subpopulations. (C) Go terms enriched in modules 9+5, 1+6, 8, 2+3 analyzed by clusterProfiler. **
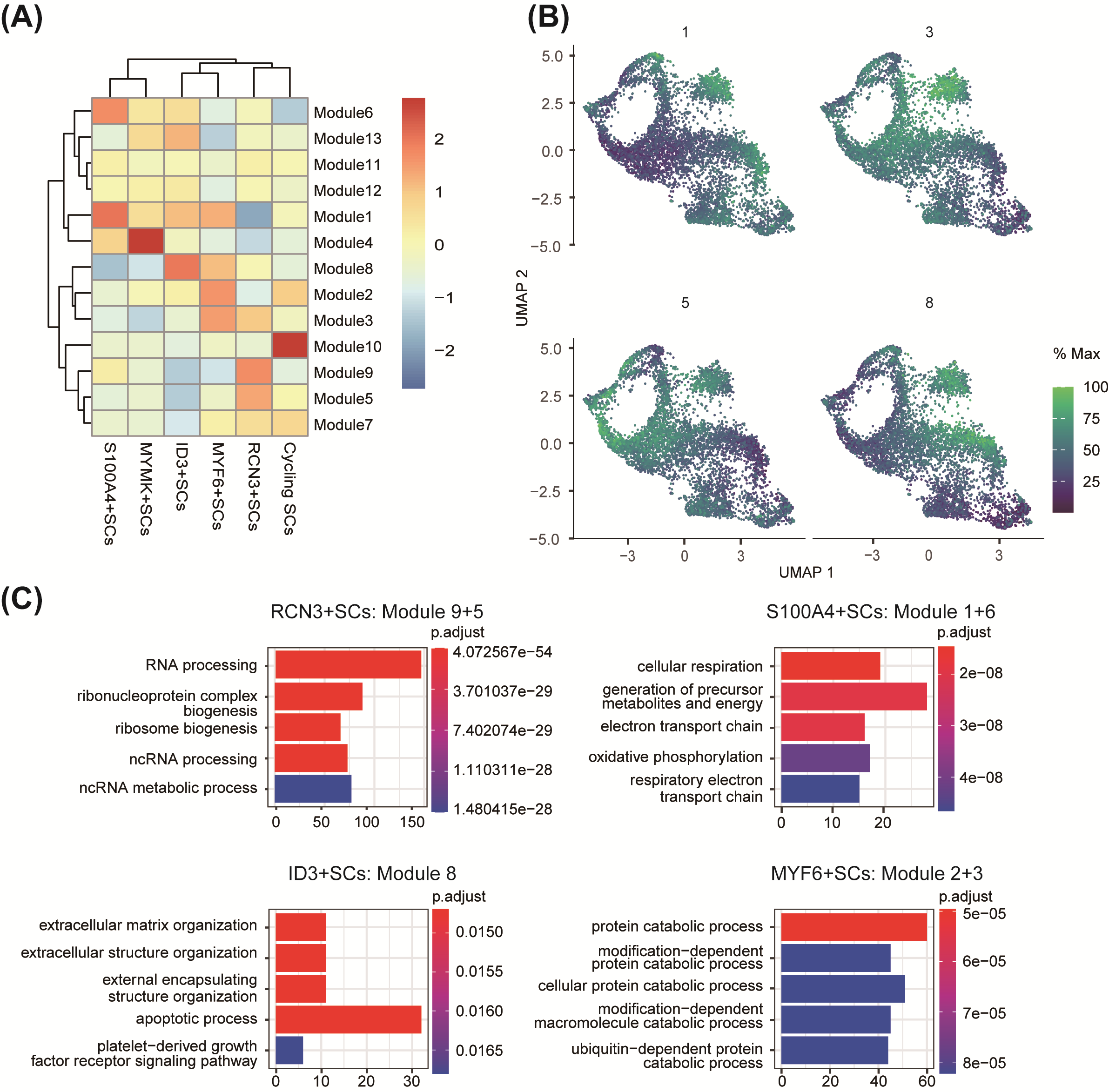
**

**Figure S4 The** **differently expressed genes in myo-lineage cells derived from LD and SOL**

(A) Heatmap of top 20 differently expressed genes in myo-lineage cells derived from LD and SOL. (B) GSEA analysis of hallmark myogenesis and oxidative phosphorylation in LD derived myo-lineage cells, and cell cycle and reactome signaling by FGFR in SOL derived myo-lineage cells.

**
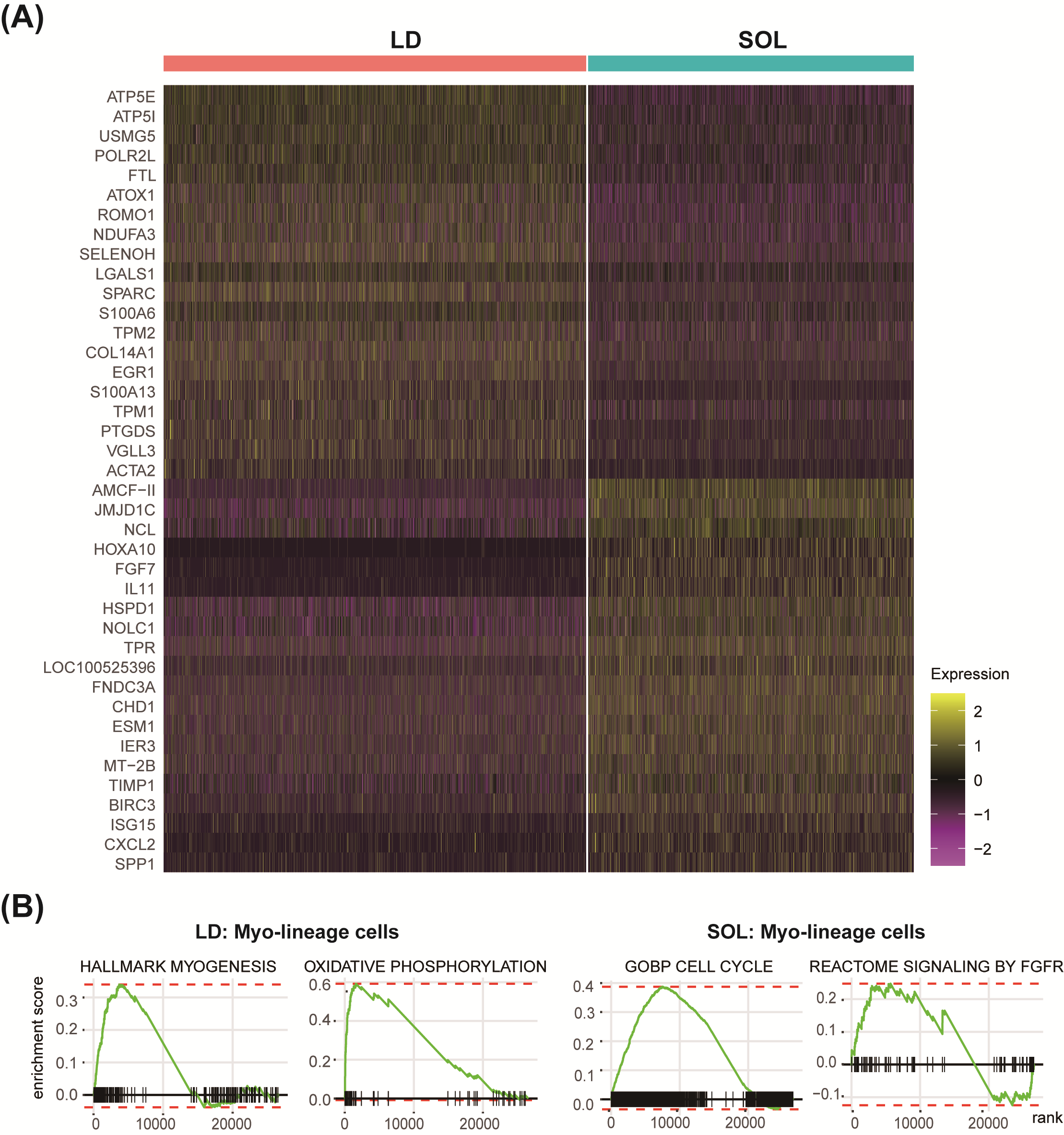
**

**Figure S5 Satellite cells derived from porcine *soleus* muscles showed better potential of proliferation**

(A) Immunofluorescence staining of Fast (red) and Slow (green) myosin heavy chain, Pax7 (green) and Ki67 (red) in 3-day-old piglets LD and SOL muscles, and the ratio of Fast and Slow fiber type and Pax7^+^ cells, Ki67^+^ **verse** Pax7^+^ cells were quantified. The scale bar of Fast+Slow and Pax7+Ki67 staining is 20 μm and 100 μm, respectively. (n = 5) (B) Immunofluorescence staining of Pax7 (red) and MyoD (green) to test the purity of cultured LD-MuSC**s** and SOL-MuSC**s** (Scale bar is 50 μm) (C) Immunofluorescence staining with EdU (red) and hoechst (blue), Ki67 (red) and DAPI (blue) in porcine LD-MuSC**s** and SOL-MuSC**s**, and the ratio of EdU^+^ cells and Ki67^+^ cells were quantified. The scale bar is 100 μm. (n = 3) (D) Western blotting technology was used to detect the levels of protein associated with cell proliferation (Ki67, Pax7, p21, CyclinD and PCNA) in LD-MuSC and SOL-MuSC, and they were quantitatively analyzed. (**P* < 0.05, ***P* < 0.01, ****P* ≤ 0.001). Data are presented as mean ± SD.


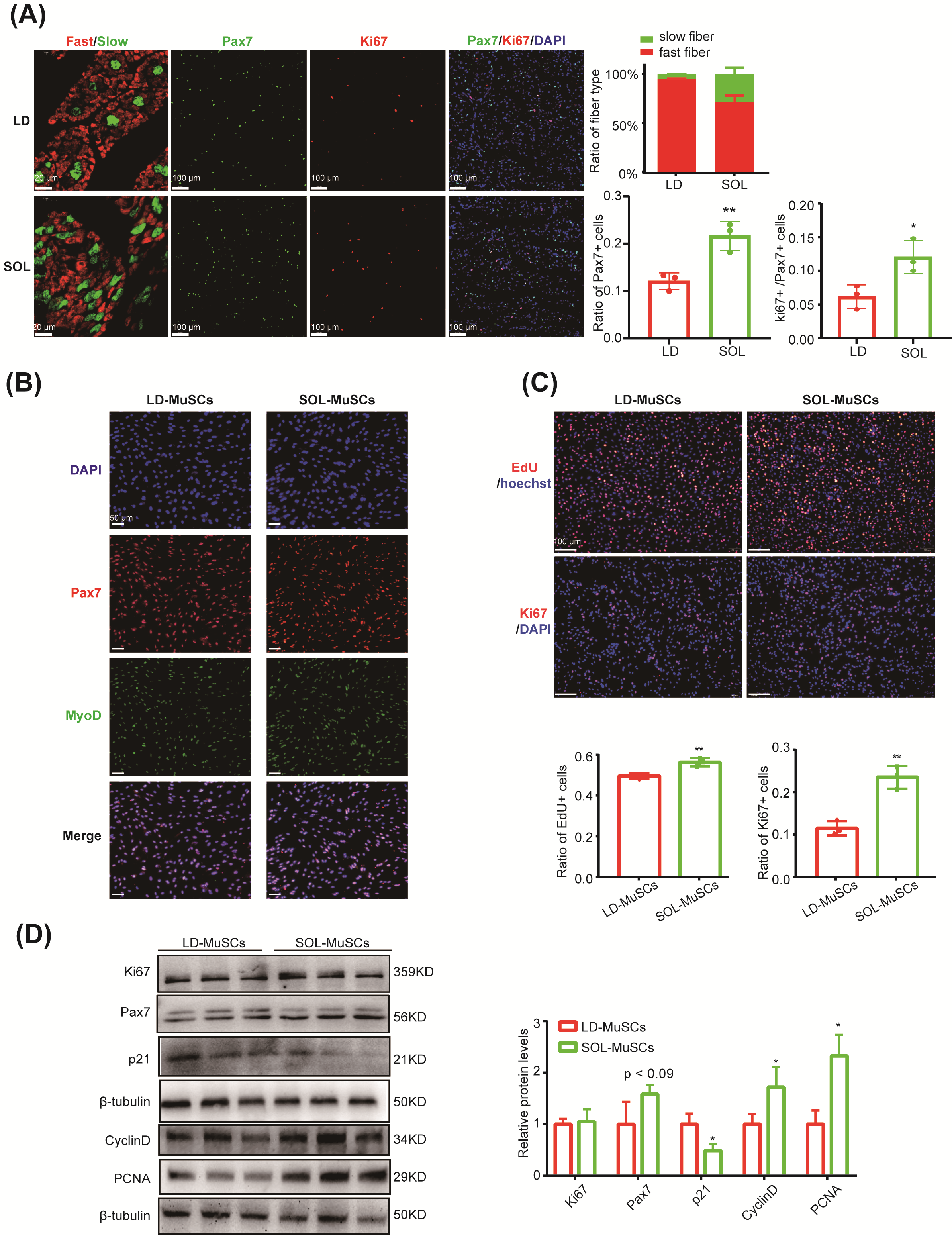


**Figure S6 Cell-cell interactions between porcine FAPs and myogenic cell subpopulations**

(A) The outgoing communication patterns of secreting cells, which showed the correspondence between the inferred latent patterns and cell groups, as well as signaling pathways. (B) The incoming communication patterns of target cells

**
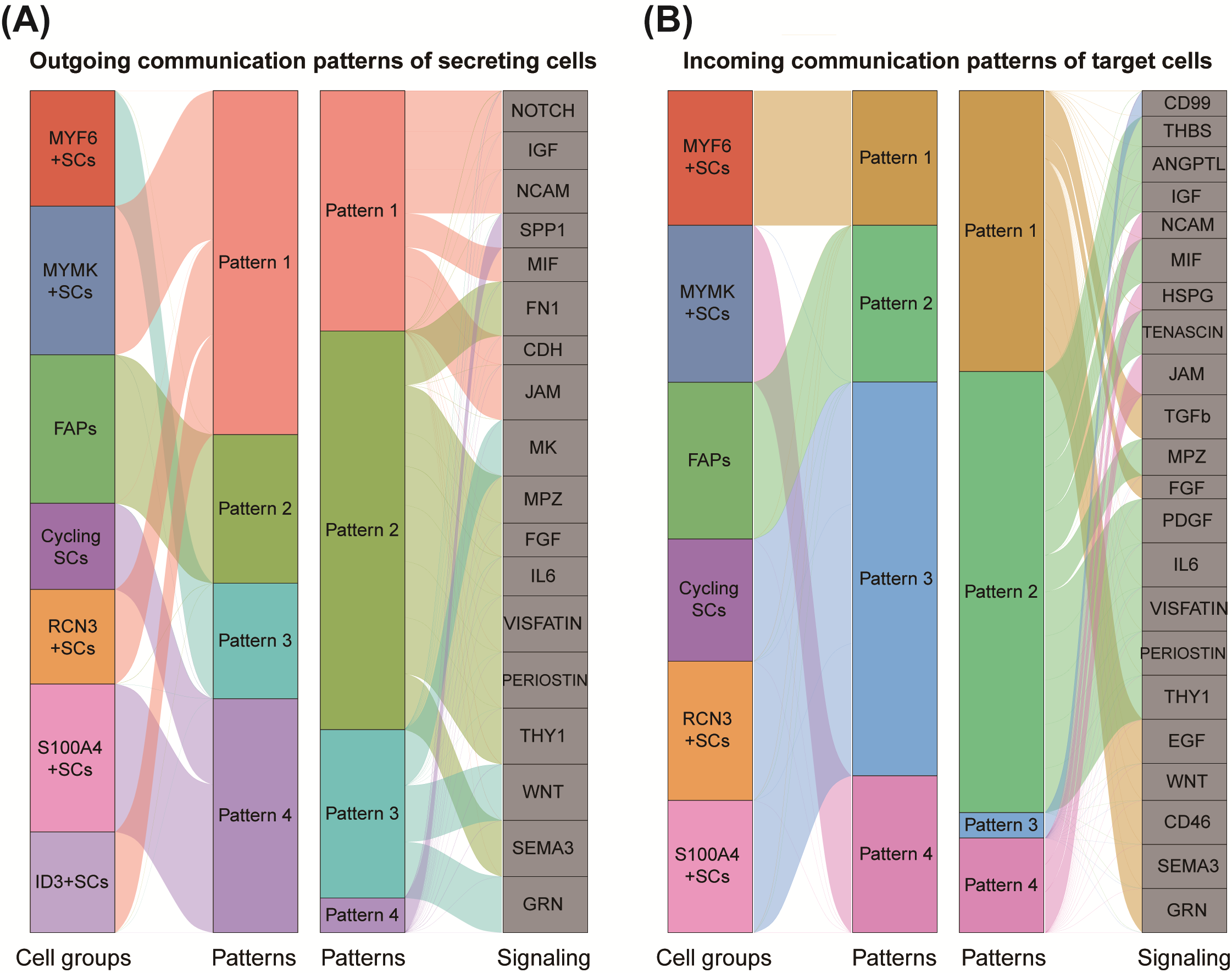
**

**Figure S7 The expression of FGFR2 in myogenic cells**

(A) Violin plots of FGFRs in porcine myogenic cell populations (B) The mRNA expression of FGFR2 in proliferating and differentiating C2C12 cells. (n = 6) (C) The mRNA expression of FGFR2 in proliferating and differentiating porcine MuSCs. (n = 6) (D) The mRNA expression of FGFR2 in proliferating LD-MuSC and SOL-MuSC. (n = 6) (E) AUCell analyses identified myo-lineage cells activated with FGF activated receptor activity and Type 2 FGFR binding gene sets. (F) Western blot analysis of p38 MAPK, p-p38 MAPK, ERK1/2 and p-ERK1/2 in C2C12 cells treated with si-pFGFR2 and 10 ng/ml FGF7 recombinant protein. The ratio of p-p38 MAPK verse p38 MAPK and p-ERK1/2 verse ERK1/2 protein levels were quantified. (**P* < 0.05, ***P* < 0.01). Data are presented as mean ± SD.


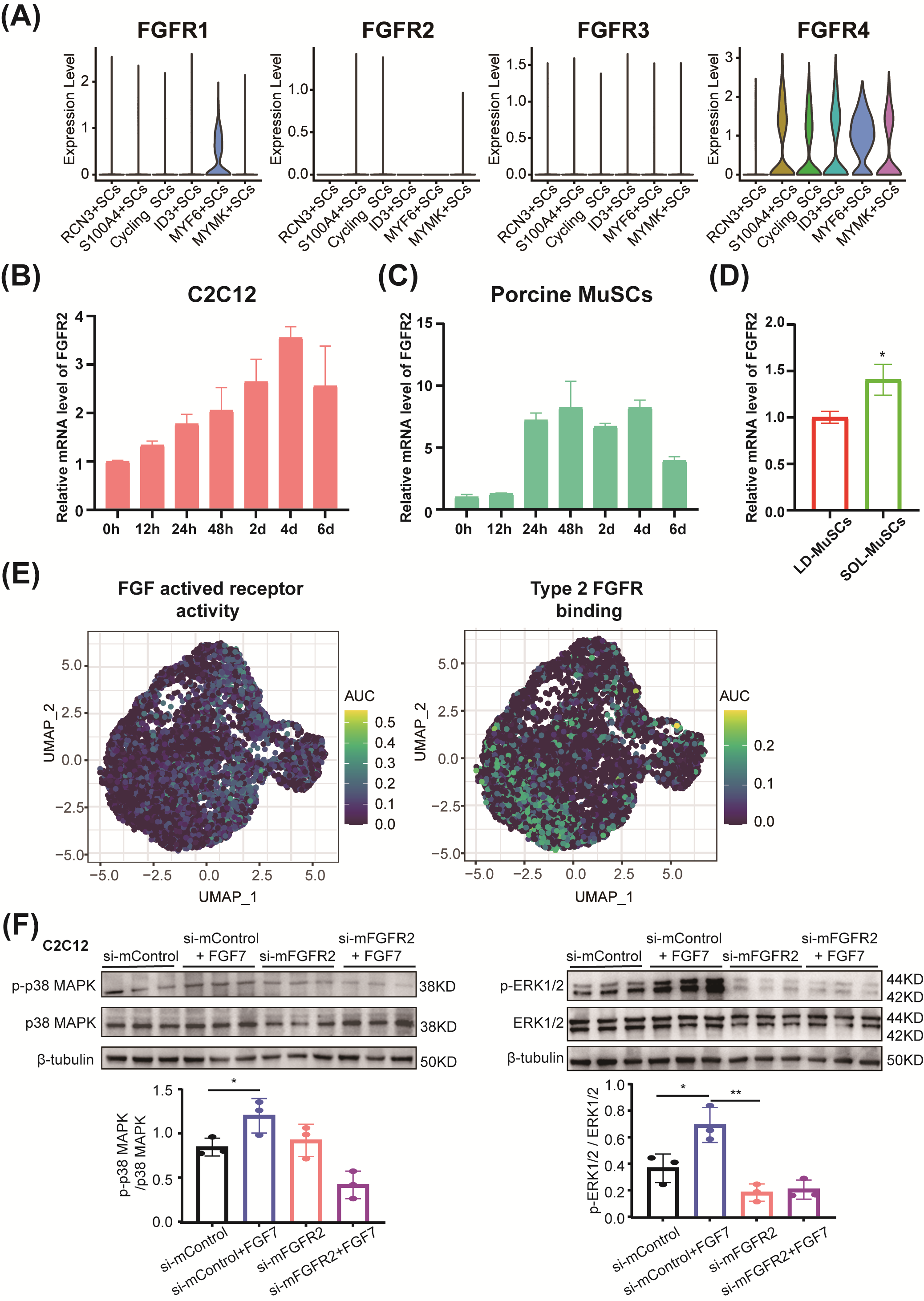


**Figure S8 FGF7 delayed myoblast senescence**

(A) Immunofluorescence staining with EdU (red) and hoechst (blue) in porcine MuSCs treated with a series of concentrations of D-gal (0, 1, 5, 10, 20, 40 g/L), and (B) the ratio of EdU^+^ cells were quantified. (n = 4) (C) CCK8 was used to detect the cell viability of porcine MuSCs treated with D-gal (0, 1, 5, 10, 20, 40 g/L). (n = 5) (D) Immunofluorescence staining with γH2AX (green) and DAPI (blue) was detected in C2C12 treated with 10 ng/ml FGF7 recombinant protein, 20 g/L D-gal, 20 g/L D-gal + 10 ng/ml FGF7, and the nuclear area (n = 200 nuclei) and ratio of γH2AX^+^ cells (n = 3) were quantified. The scale bar of (A) is 100 μm and (D) is 10 μm. (**P* < 0.05, ***P* < 0.01, ****P* ≤ 0.001). Data are presented as mean ± SD.

**
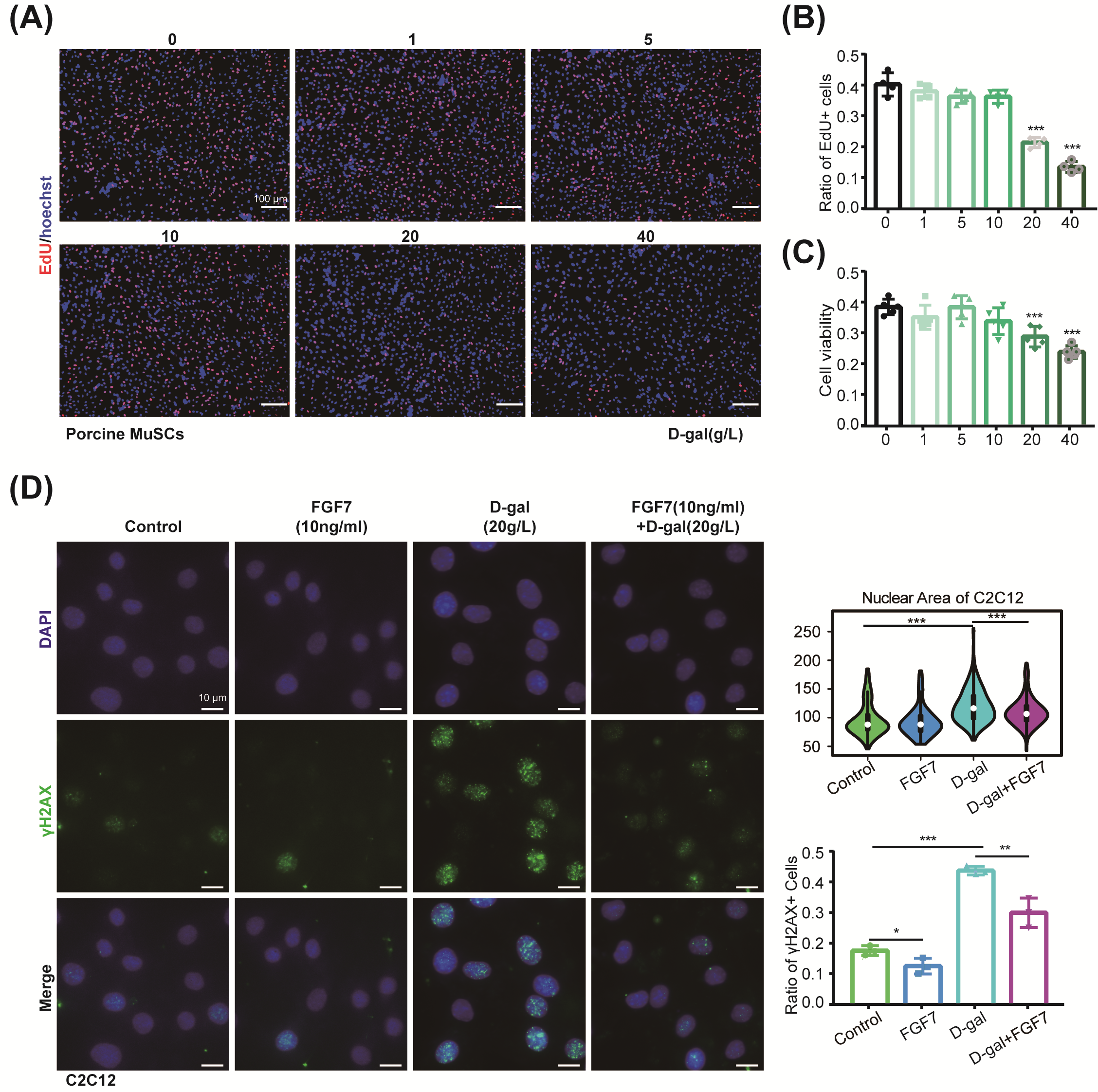
**

**Table S1: Primary antibodies used in this study.**

| Antibody | Dilution (WB/IF) | Source | Cat. No |
| --- | --- | --- | --- |
| MyHC-fast | no/1:100 | Sigma-Aldrich | M4276 |
| MyHC-slow | no/1:100 | Sigma-Aldrich | MABT849 |
| Ki67 | 1:1000/1:100 | Servicebio | GB111499 |
| PAX7 | 1:1000/no | Abcom | CSB-PA017493ESR1HU |
| P21 | 1:1000/no | Abways | CY5088 |
| CyclinD | 1:1000/no | Abways | CY5404 |
| PCNA | 1:1000/no | Santa Cruz | Sc-56 |
| β-tubulin | 1:1000/no | Abways | AB0057 |
| FGF7 | 1:1000/no | Novus | NBP1-91898 |
| FGFR2 | No/1:100 | Abways | CY6723 |
| eMyHC | no/1:100 | Bioss | bs-10905R |
| Pax7 | no/1:100 | Abcom | ab187339 |
| MyoD | no/1:100 | Santa Cruz | Sc-377460 |
| p38 MAPK | 1:1000/no | Wanleibio | WL00764 |
| p-p38 MAPK | 1:500/no | Wanleibio | WLP1576 |
| ERK1/2 | 1:1000/no | Vazyme | RA2201 |
| p-ERK1/2 | 1:500/no | Vazyme | RA2202 |
| Laminin β-2 (C4) | no/1:100 | Santa Cruz | Sc-59980 |
| γH2AX | 1:1000/no | FineTest | FNab10441 |
| Goat anti-rabbit (HRP) | 1:3000/no | Sigma-Aldrich | A21020 |
| Goat anti-mouse (HRP) | 1:3000/no | Sigma-Aldrich | A21021 |
| Goat anti-rabbit (Alexa Fluor 546) | no/1:1000 | Invitrogen | A-11035 |
| Goat anti-mouse (Alexa Fluor 546) | no/1:1000 | Invitrogen | A-11003 |
| Goat anti-mouse (FITC) | no/1:1000 | Transgene | HS211-01 |

**Abbreviations**

UMAP, Uniform Manifold Approximation and Projection; GSEA, Gene Set Enrichment Analysis; PAX7, paired box 7; CD82, CD82 molecule; MYMK, myomaker, myoblast fusion factor; ITGA7, integrin subunit alpha 7; CDKN1C, cyclin dependent kinase inhibitor 1C; PDGFRA, platelet derived growth factor receptor alpha; PDGFRB, platelet derived growth factor receptor beta; FGF7, fibroblast growth factor 7; TIMP3, TIMP metallopeptidase inhibitor 3; CDH5, cadherin 5; PECAM1, platelet and endothelial cell adhesion molecule 1; ECSCR, endothelial cell surface expressed chemotaxis and apoptosis regulator; COMP, cartilage oligomeric matrix protein; THBS4, thrombospondin 4; TNMD, tenomodulin; COL11A1, collagen type XI alpha 1 chain; FMOD, fibromodulin; C1QA, complement C1q A chain; C1QB, complement C1q B chain; C1QC, complement C1q C chain; MMP9, matrix metallopeptidase 9; MMP3, matrix metallopeptidase 3; CENPF, centromere protein F; NUMB, NUMB endocytic adaptor protein; S100A11, S100 calcium binding protein A11; ID3, inhibitor of DNA binding 3; FGFR, fibroblast growth factor receptor; PCNA, proliferating cell nuclear antigen; CD99, CD99 molecule (Xg blood group); IGF, insulin like growth factor; MIF, macrophage migration inhibitory factor; SPP1, secreted phosphoprotein 1; PDGF, platelet derived growth factor; ANGPTL, angiopoietin like; FN1, fibronectin 1; MPZ, myelin protein zero; THY1, Thy-1 cell surface antigen; IL6, insulin like growth factor 6; SEMA3, semaphorin 3; HSPG, heparan sulfate proteoglycans; NCAM, neural cell adhesion molecule; FGFR2, fibroblast growth factor receptor 2; γH2AX, phosphorylated form of histone variant H2AX; RCN3, [reticulocalbin 3](https://www.ncbi.nlm.nih.gov/gene/57333); FSTL3, follistatin like 3; S100A4, S100 calcium binding protein A4; MYF6, myogenic factor 6; FGF6, fibroblast growth factor 6; EGF, epidermal growth factor; CD34, CD34 molecule; ID4, inhibitor of DNA binding 4; JAM, junctional adhesion molecule; MKI67, marker of proliferation Ki-67; CDH, cadherin; GRN, granulin precursor; TGFb, transforming growth factor-β; MAPK, mitogen activated protein kinase; ERK, extracellular signal-regulated kinase; MYF5, myogenic factor 5; CSA, cross-sectional area; CTX, cardiotoxin; SDF1α, stromal cell-derived factor-1α; G-CSF, granulocyte colony-stimulating factor; CXCR4, C-X-C motif chemokine receptor 4; WISP1, WNT1 inducible signaling pathway protein 1; MYOG, myogenin; ID2, inhibitor of DNA binding 2; MYOD1, myogenic differentiation 1.

**Supplemental Methods**

**Single cell RNA data analysis**

Raw counts matrices were filtered by selecting cells expressing 200-6000 genes with less than 8% mitochondrial content. Genes exhibiting expression in fewer than three cells were also excluded from the following analysis. A total of 16270 cells were collected by Cell Ranger package (10X Genomics). An average of 39124 reads and 2315 genes were detected per cell. Doublets were evaluated and removed with ScdblFinder 1.15.3 [S6, S7]. After filtering the doublets, a total of 14358 cells (5791 cells from LD and 8567 cells from SOL) were used to create Seurat object and following analysis using Seurat 4.3.0 in R 4.1.1 and normalized with "LogNormalize" method [S5]. Dimension reduction was conducted by Uniform Manifold Approximation and Projection (UMAP) method of Seurat. The integration of data from different samples were performed using Harmony 0.1.1 [S8]. To determine the best clustering method, the FindClusters method of Seurat was performed and resolution 0.5 was selected. Differential expressed genes of each cluster were analyzed by the FindAllMarkers function based on the following parameters: logfc.threshold = 0.25, min.pct = 0.25, and min.diff.pct = 0.25. Combined with the classical genes reported from the literature, 14358 cells were defined into 5 cell types. In addition, the clustering of myo-lineage cells was performed with UMAP method of Seurat in resolution 0.3. Pseudo-temporal analysis was conducted with monocle3, and the co-expression module analysis was performed using the “gene_module_df” function in resolution 0.001 [S9]. RNA velocity analysis was performed with all SCs derived from LD and SOL using scVelo 0.2.5 package in python 3.9.1 [S10].

The gene set enrichment score analysis (GSEA) of pathways enriched in myo-lineage cells, FAPs, and LD or SOL derived myo-lineage cells were performed with fgsea 1.18.0 package [S41]. The database was C5 ontology gene sets from molecular signatures database (MSigDB) on the GSEA website (<http://software.broadinstitute.org/gsea/msigdb>). The Gene Ontology (GO) analysis of genes in different myo-lineage cell modules was performed using clusterProfiler 4.0.5.

AUCell (1.14.0) algorithm was used to identify cells with active genes in scRNA data [S12]. Several published data (GSE103164, GSE188215) were used as the input gene sets of AUCell analysis. In addition, the input gene sets of FGFR signaling used for AUCell was selected from MSigDB database.

Cellchat 1.6.1 (R 4.3.1) was performed to infer all cell-cell communications across all cell types [S13]. The communication pattern analysis was employed to identify the outgoing and incoming pattern of each signaling pathway. And the information flow differences between LD and SOL was determined by rankNet method based on paired Wilcoxon test.

**Porcine muscle satellite cell isolation**

Porcine muscle satellite cells (MuSCs) were isolated from 3-day-old Duroc × Landrace × Yorkshire littermate piglets which were obtained from the Third Experimental Farm of Northwest A&F University, Yangling, China. MuSCs were isolated and cultured as described previously [S42, S43]. Piglets were anesthetized with sodium pentobarbital, and sacrificed with 10% KCl (0.5mL/kg of body weight) via an otogenic vein. *Longissimus dorsi* and *soleus* muscle tissues were collected and subsequent experiments were carried out simultaneously. Collected tissues were stored in phosphate-buffered saline (PBS; Invitrogen, Carlsbad, CA, USA) containing 5% penicillin and streptomycin (PB180120, Procell). Porcine MuSCs were obtained by two-step enzyme digestion process and multiple differential adhesion method. Brifely, muscle tissues were digested with 1mg/ml college type II (Invitrogen) for 40 minutes at 37°C, and 0.25% trypsin (ThermoFisher scientific) for 30 minutes at 37°C. 10% fetal bovine serum (FBS) (Hyclone, Connecticut, USA) was used to terminate the digestion process. Primary cell suspensions were obtained by filtering through 70 and 200 mesh strainers. The cells were then washed up by RPMI medium 1640 basic (ThermoFisher scientific, A10491-01) for twice, and subsequently seeded in cell culture plates for adherence and growth.

**Real-time quantitative PCR (RT–qPCR)**

Total RNA was extracted from cells using TRIzol reagent (Invitrogen, Carlsbad, CA). cDNA was synthesized with reverse transcription kits (TaKaRa, Otsu, Japan). RT–qPCR was performed by RT–qPCR using SYBR Green (Vazyme, Nanjing, China) in the Step One Plus system (ABI, MA, USA). The procedure of RT–qPCR was as follow: 3 min at 95°C, 40 cycles of 10s at 95°C and 30s at 60°C. The Ct (2^−ΔΔCt^) method was used to calculate the relative RNA expression [S44]. The RT–qPCR primer sequences of porcine FGFR2 were F: 5’-GTGATGTCTGGTCCTTCGGG-3’ and R: 5’-GTTCGTTGGTGCAGTTTGCT-3’, and the primer sequences of reference gene were porcine GAPDH (F: 5’-AGGTCGGAGTGAACGGATTTG-3’; R: 5’-ACCATGTAGTGGAGGTCAATGAAG-3’). The qPCR primer sequences of mice FGFR2 were F: 5’-TGACATTAACCGTGTTCCTGAG-3’ and R: 5’- TGACATTAACCGTGTTCCTGAG-3’, and the sequences of reference gene were mice GAPDH (F: 5’-CCCAGAAGACTGTGGATGG-3’; R: 5’-ACACATTGGGGGTAGGAACA-3’).

**Cell viability assay**

Cell viability of porcine MuSCs was determined with CCK8 (cell counting kit 8) kit (MI00615A, MI00615A, mishushengwu, China). MuSCs were seeded into 96-well plates at 2×10^3^ cells per well, and incubated with CCK8 reagent at 37 ℃ for 4 hours after treatment. The absorbance was measured at 450 nm.

**Mouse treadmill exhaustion test**

Treadmill exhaustion test was performed for D-gal-induced aging mice after three injections of FGF7 (experimental groups: control, D-gal, D-gal + FGF7), according to previous studies [S18, S19]. Mice were first familiarized with 30 min at 10 m/min for 3 days on a motorized treadmill. And then mice were tested by running at 10 m/min for 20 min, while the speed was increased by 2 m/min every 20 min until the mice were considered exhausted. Exhaustion termination was confirmed when the mice sat on the shock grid behind the treadmill for more than 20 s. The distance and time running to exhaustion were recorded and calculated for all mice.

**Immunofluorescence analysis**

Whole-slide digital images were collected with an Pannoramic DESK Scanner (P-MIDI, P250, 3D HISTECH). The calculation of porcine fast and slow myofiber types and Pax7^+^ nucleus to myofibers were performed with ImageJ for approximately 5 fields of each view, and 5 views for each slice. The ratio of EdU-positive nucleus to total nucleus was calculated for 200 nuclei per sample using ImageJ. The eMyHC^+^ area to total area was measured with aipathwell (Servicebio) software. The myofiber CSA showed with Laminin immunofluorescence staining was calculated using ImageJ for approximately 5 fields of view for each slice.

The images of immunostaining with Ki67 in cultured cells were acquired using BioTEK gen5 Software. Ki67^+^ nucleus and total cell nucleus were counted using ImageJ. The images of immunostaining with γH2AX and DAPI in cultured cells were acquired using confocal laser scanning microscopy (Revolution WD; Andor). The nuclear area of DAPI were measured with ImageJ for approximately 200 nuclei per sample. And γH2AX^+^ nucleus numbers were counted with ImageJ for approximately 200 nuclei per sample.

**Supplemental References**

S1. Li H, Chen Q, Li C, Zhong R, Zhao Y, Zhang Q, et al. Muscle-secreted granulocyte colony-stimulating factor functions as metabolic niche factor ameliorating loss of muscle stem cells in aged mice. Embo j 2019;38:e102154.

S2. Shams AS, Arpke RW, Gearhart MD, Weiblen J, Mai B, Oyler D, et al. The chemokine receptor CXCR4 regulates satellite cell activation, early expansion, and self-renewal, in response to skeletal muscle injury. Front Cell Dev Biol. 2022;10:949532.

S3. Xi H, Langerman J, Sabri S, Chien P, Young CS, Younesi S, et al. A Human Skeletal Muscle Atlas Identifies the Trajectories of Stem and Progenitor Cells across Development and from Human Pluripotent Stem Cells. Cell stem cell 2020;27:181–185.

S4. Yartseva V, Goldstein LD, Rodman J, Kates L, Chen MZ, Chen Y-JJ, et al. Heterogeneity of Satellite Cells Implicates DELTA1/NOTCH2 Signaling in Self-Renewal. Cell Rep 2020;30: 1491–1503.e1496.

S5. Xi NM, Li JJ. Protocol for executing and benchmarking eight computational doublet-detection methods in single-cell RNA sequencing data analysis. STAR Protoc 2021;2:100699.

S6. Butler A, Hoffman P, Smibert P, Papalexi E, Satija R. Integrating single-cell transcriptomic data across different conditions, technologies, and species. Nat Biotechnol 2018;36:411–420.

S7. Germain PL, Lun A, Garcia Meixide C, Macnair W, Robinson MD. Doublet identification in single-cell sequencing data using scDblFinder. F1000Res 2021;10:979.

S8. Korsunsky I, Millard N, Fan J, Slowikowski K, Zhang F, Wei K, et al. Fast, sensitive and accurate integration of single-cell data with Harmony. Nat Methods 2019;16:1289–1296.

S9. Cao J, Spielmann M, Qiu X, Huang X, Ibrahim DM, Hill AJ, et al. The single-cell transcriptional landscape of mammalian organogenesis. Nature 2019;566:496–502.

S10. La Manno G, Soldatov R, Zeisel A, Braun E, Hochgerner H, Petukhov V, et al. RNA velocity of single cells. Nature 2018;560:494–498.

S11. Hänzelmann S, Castelo R, Guinney J. GSVA: gene set variation analysis for microarray and RNA-seq data. BMC Bioinformatics 2013;14:7.

S12. Aibar S, González-Blas CB, Moerman T, Huynh-Thu VA, Imrichova H, Hulselmans G, et al. SCENIC: single-cell regulatory network inference and clustering. Nat Methods 2017;14:1083–1086.

S13. Jin S, Guerrero-Juarez CF, Zhang L, Chang I, Ramos R, Kuan CH, et al. Inference and analysis of cell-cell communication using CellChat. Nat Commun 2021;12:1088.

S14. Chen QN, Fan Z, Lyu AK, Wu J, Guo A, Yang YF, et al. Effect of sarcolipin-mediated cell transdifferentiation in sarcopenia-associated skeletal muscle fibrosis. Exp Cell Res 2020;389:111890.

S15. Yang YF, Yang W, Liao ZY, Wu YX, Fan Z, Guo A, et al. MICU3 regulates mitochondrial Ca(2+)-dependent antioxidant response in skeletal muscle aging. Cell Death Dis 2021;12:1115.

S16. Shi XC, Xia B, Zhang JF, Zhang RX, Zhang DY, Liu H, et al. Optineurin promotes myogenesis during muscle regeneration in mice by autophagic degradation of GSK3β. PLoS Biol 2022;20:e3001619.

S17. Shi D, Gu R, Song Y, Ding M, Huang T, Guo M, et al. Calcium/Calmodulin-Dependent Protein Kinase IV (CaMKIV) Mediates Acute Skeletal Muscle Inflammatory Response. Inflammation 2018;41:199–212.

S18. Janice Sánchez B, Tremblay AK, Leduc-Gaudet JP, Hall DT, Kovacs E, Ma JF, et al. Depletion of HuR in murine skeletal muscle enhances exercise endurance and prevents cancer-induced muscle atrophy. Nat Commun 2019;10:4171.

S19. Song H, Tian X, Liu D, Liu M, Liu Y, Liu J, et al. CREG1 improves the capacity of the skeletal muscle response to exercise endurance via modulation of mitophagy. Autophagy 2021;17:4102–4118.

S20. Ma L, Tian X, Xi F, He Y, Li D, Sun J, et al. Ablation of Tas1r1 Reduces Lipid Accumulation Through Reducing the de Novo Lipid Synthesis and Improving Lipid Catabolism in Mice. J Agric Food Chem 2022;70:10248–10258.

S21. Alexander MS, Rozkalne A, Colletta A, Spinazzola JM, Johnson S, Rahimov F, et al. CD82 Is a Marker for Prospective Isolation of Human Muscle Satellite Cells and Is Linked to Muscular Dystrophies. Cell stem cell 2016;19:800–807.

S22. Giordani L, He GJ, Negroni E, Sakai H, Law JYC, Siu MM, et al. High-Dimensional Single-Cell Cartography Reveals Novel Skeletal Muscle-Resident Cell Populations. Mol Cell 2019;74:609–621.e606.

S23. Choi Y, Shin S, Son HJ, Lee NH, Myeong SH, Lee C, et al. Identification of potential biomarkers related to mesenchymal stem cell response in patients with Alzheimer's disease. Stem Cell Res Ther 2023;14:178.

S24. Do NT, Lee SY, Lee YS, Shin C, Kim D, Lee TG, et al. Time-sequential fibroblast-to-myofibroblast transition in elastin-variable 3D hydrogel environments by collagen networks. Biomater Res 2023;27:103.

S25. Bruijn LE, van den Akker B, van Rhijn CM, Hamming JF, Lindeman JHN. Extreme Diversity of the Human Vascular Mesenchymal Cell Landscape. J Am Heart Assoc 2020;9:e017094.

S26. Shinin V, Gayraud-Morel B, Tajbakhsh S. Template DNA-strand co-segregation and asymmetric cell division in skeletal muscle stem cells. Methods Mol Biol 2009;482:295–317.

S27. Wang J, Broer T, Chavez T, Zhou CJ, Tran S, Xiang Y, et al. Myoblast deactivation within engineered human skeletal muscle creates a transcriptionally heterogeneous population of quiescent satellite-like cells. Biomaterials 2022;284:121508.

S28. Shirakawa T, Toyono T, Inoue A, Matsubara T, Kawamoto T, Kokabu S. Factors Regulating or Regulated by Myogenic Regulatory Factors in Skeletal Muscle Stem Cells. Cells 2022;11.

S29. Murgia M, Toniolo L, Nagaraj N, Ciciliot S, Vindigni V, Schiaffino S, et al. Single Muscle Fiber Proteomics Reveals Fiber-Type-Specific Features of Human Muscle Aging. Cell Rep 2017;19:2396–2409.

S30. Yablonka-Reuveni Z, Danoviz ME, Phelps M, Stuelsatz P. Myogenic-specific ablation of Fgfr1 impairs FGF2-mediated proliferation of satellite cells at the myofiber niche but does not abolish the capacity for muscle regeneration. Front Aging Neurosci 2015;7:85.

S31. Wu Y, Zhang X, Salmon M, Lin X, Zehner ZE. TGFbeta1 regulation of vimentin gene expression during differentiation of the C2C12 skeletal myogenic cell line requires Smads, AP-1 and Sp1 family members. Biochim Biophys Acta 2007;1773:427–439.

S32. Zhu X-J, Yuan X, Wang M, Fang Y, Liu Y, Zhang X, et al. A Wnt/Notch/Pax7 signaling network supports tissue integrity in tongue development. J Biol Chem 2017;292:9409–9419.

S33. Ghadiali RS, Guimond SE, Turnbull JE, Pisconti A. Dynamic changes in heparan sulfate during muscle differentiation and ageing regulate myoblast cell fate and FGF2 signalling. Matrix Biol 2017;59:54–68.

S34. Lin YH, Chou LY, Chou HC, Chen CH, Kang L, Cheng TL, et al. The Essential Role of Stathmin in Myoblast C2C12 for Vertical Vibration-Induced Myotube Formation. Biomolecules 2021;11.

S35. Kästner S, Elias MC, Rivera AJ, Yablonka-Reuveni Z. Gene expression patterns of the fibroblast growth factors and their receptors during myogenesis of rat satellite cells. J Histochem Cytochem 2000;48:1079–1096.

S36. Chen X, Ouyang H, Wang Z, Chen B, Nie Q. A Novel Circular RNA Generated by FGFR2 Gene Promotes Myoblast Proliferation and Differentiation by Sponging miR-133a-5p and miR-29b-1-5p. Cells 2018;7.

S37. Zinkle A, Mohammadi M. A threshold model for receptor tyrosine kinase signaling specificity and cell fate determination. F1000Res 2018;7.

S38. Liu S, Sun D, Butler R, Rawlins EL. RTK signalling promotes epithelial columnar cell shape and apical junction maintenance in human lung progenitor cells. Development 2023;150.

S39. Elabd C, Cousin W, Upadhyayula P, Chen RY, Chooljian MS, Li J, et al. Oxytocin is an age-specific circulating hormone that is necessary for muscle maintenance and regeneration. Nat Commun 2014;5:4082.

S40. Berent-Maoz B, Montecino-Rodriguez E, Signer RA, Dorshkind K. Fibroblast growth factor-7 partially reverses murine thymocyte progenitor aging by repression of Ink4a. Blood 2012;119:5715–5721.

S41. Korotkevich G, Sukhov V, Budin N, Shpak B, Artyomov MN, Sergushichev A. Fast gene set enrichment analysis. 2021:060012.

S42. Lv W, Jin J, Xu Z, Luo H, Guo Y, Wang X, et al. lncMGPF is a novel positive regulator of muscle growth and regeneration. J Cachexia Sarcopenia Muscle 2020;11:1723–1746.

S43. Yue F, Bi P, Wang C, Li J, Liu X, Kuang S. Conditional Loss of Pten in Myogenic Progenitors Leads to Postnatal Skeletal Muscle Hypertrophy but Age-Dependent Exhaustion of Satellite Cells. Cell Rep 2016;17:2340–2353.

S44. Livak KJ, Schmittgen TD. Analysis of relative gene expression data using real-time quantitative PCR and the 2(-Delta Delta C(T)) Method. Methods 2001;25:402–408.
